# Genetic Aspects and Molecular Causes of Seed Longevity in Plants—A Review

**DOI:** 10.3390/plants11050598

**Published:** 2022-02-23

**Authors:** Mian Abdur Rehman Arif, Irfan Afzal, Andreas Börner

**Affiliations:** 1 Wheat Breeding Group, Plant Breeding and Genetics Division, Nuclear Institute for Agriculture and Biology, Faisalabad 38000, Pakistan; 2 Seed Physiology Lab, Department of Agronomy, University of Agriculture, Faisalabad 38000, Pakistan; irfanuaf@gmail.com; 3 Leibniz-Institute für Pflanzengenetik und Kulturpflanzenforschung (IPK), OT Gatersleben, D-06466 Seeland, Germany

**Keywords:** seed longevity, genetics, quantitative trait loci, candidate genes, genebanks

## Abstract

Seed longevity is the most important trait related to the management of gene banks because it governs the regeneration cycle of seeds. Thus, seed longevity is a quantitative trait. Prior to the discovery of molecular markers, classical genetic studies have been performed to identify the genetic determinants of this trait. Post-2000 saw the use of DNA-based molecular markers and modern biotechnological tools, including RNA sequence (RNA-seq) analysis, to understand the genetic factors determining seed longevity. This review summarizes the most important and relevant genetic studies performed in Arabidopsis (24 reports), rice (25 reports), barley (4 reports), wheat (9 reports), maize (8 reports), soybean (10 reports), tobacco (2 reports), lettuce (1 report) and tomato (3 reports), in chronological order, after discussing some classical studies. The major genes identified and their probable roles, where available, are debated in each case. We conclude by providing information about many different collections of various crops available worldwide for advanced research on seed longevity. Finally, the use of new emerging technologies, including RNA-seq, in seed longevity research is emphasized by providing relevant examples.

## 1. Introduction

Sustainable agriculture depends on the judicial use of natural resources, including use of crop varieties that are resistant to pests and diseases and do not require pesticide spraying, thus providing environmental benefits in addition to fulfilling the energy requirements of mankind. At present, >7000 plants are cultivated for food, shelter, and other purposes [1], and approximately 50% of human food comes from maize, wheat, and rice. Climate change has posed an extinction threat to 8% of the 250,000 species of flowering plants by 2025. To arrest this calamity, plant genetic resources are stored and regenerated in >1750 gene banks storing >7,000,000 accessions [2]. To successfully maintain such an extensive germplasm, a systematic evaluation of seed survival and longevity of the plant material stored is always under progress [3]. Because seeds are the prime storage material, research on seed longevity is of particular importance [4].

Seed longevity is defined as the maximum time period during which seeds can germinate [5,6,7] and produce viable seedlings capable of developing into healthy plants and bearing seeds for the next generation. In addition to many other features, seed longevity is influenced by pre-storage and storage conditions and the genetic and physiological storage potential of seeds. It is also affected by harsh conditions during or after seed development or damage prior to or during storage [8]. The long-term storage of seeds, particularly under unfavorable conditions, leads to the loss of viability, which is variable in nature. Loss of viability is related to various seed properties, including color, weight, and membrane composition, which are often species or, in some cases, even variety specific [9].

Seed quality can be reduced in parental plants owing to adverse environmental conditions, premature germination [10] and pathogens [11]. Damage to seed quality can be categorized into either short-term deterioration (occurring in the field, such as deterioration of the mother plant) or long-term deterioration (occurring during storage). The latter includes membrane and genetic damage, changes in respiratory activity and enzymes and protein damage [9,10]. All parts of the seed deteriorate with time, the damage from which can be sustained by the chemical constituents of seeds and the way these compounds interact to form biological structures. Integrity of DNA, proteins, and membranes is especially important for maintaining seed viability [12].

Seed deterioration during storage may involve many physical and chemical changes, including disrupted intracellular integrity, decreased enzyme activity, lipid peroxidation, and nonenzymatic reactions [13,14]. Seed viability and vigor are dependent on the integrity of cellular macromolecules and orderly compartmentalization of the cell [13]. Aging is an inexorable trend to disorder. Defense mechanisms innate to the seed’s structural and chemical features that are characteristic of a particular species may limit the rate of this decay [15].

Seed deterioration varies between different varieties of the same species. Even within a variety, the storage potential of individual lots varies, and within a seed lot, individual seeds have different storage potentials [9]. Broken, cracked, or bruised seeds deteriorate more rapidly than undamaged seeds [14,16]. Environmental stresses, including deficiency of minerals (including nitrogen, potassium, and calcium) [17]), water [18] and temperature extremes [19] during seed development and prior to physiological maturity can also reduce the longevity of seeds.

## 2. Mechanisms of Seed Ageing

A number of mechanisms of seed aging have been identified [20], including lipid peroxidation, which results in membrane damage and generation of toxic byproducts [21], oxidative damage to DNA and proteins [22], and loss of protein function during deterioration as a result of the formation of sugar–protein adducts [23]. In contrast, antioxidants, heat shock proteins (HSPs) and enzymes to repair protein damage are thought to be involved in mitigating the effects of ageing on seed longevity [24,25].

Genetic differences between species are also responsible for differential seed longevity; for example, seeds of *Canna* [26] and *Lotus* [27] are thought to be viable even after 1300 years. *Albizia* benth., *Cassia* L., *Goodia*, and *Trifolium* L. seeds can germinate after 100 years [28]. Seeds of other species are characteristically short-lived, including lettuce (*Lactuca sativa* L.), onions (*Allium cepa* L.), parsnips (*Pastinaca sativa* L.), and rye (*Secale cereal* L.) [29]. The influence of oil content on longevity under open storage conditions has also been addressed [29]; however, further analysis is required to arrive at a definitive conclusion. Species with similar chemical composition could also have significantly different storability. For example, chewing fescue (*Festuca rubra* subsp. *commutata* Gauidin) and annual ryegrass seeds have similar appearances and chemical compositions. However, ryegrass seeds were stored much better under comparable conditions [30]. Seed longevity can vary by as much as seven-fold depending on the genetic differences among cultivars of the same species [31].

Very little is known about the genetic basis of differences in seed quality because this trait is strongly affected by environmental factors during seed formation, harvest, and storage and is probably controlled by many genes. Therefore, seed longevity is a composite trait because in genetic studies of longevity [32], genetically identical seed lots of seeds, even when grown under identical conditions or derived from a single plant, lose their viability at different intervals after harvest. Seed longevity is a quantitative trait [12] and is strongly affected by the environment during seed formation, harvest, and storage [33,34] through a variety of mechanisms, whose understanding might enable us to greatly increase the seed longevity of agriculturally important species and varieties and preserve plant genetic resources for generations [35].

This review focuses on the genetic determinants of seed longevity in a variety of plant species after describing classical genetic studies.

## 3. Classical Genetic Studies

Awareness of seed longevity dates back to ~2500 years ago (372 BC–287 BC) when Theophrastus discussed seed deterioration in his botanical writings [14,36] Similarly, in Fan Sheng-zhi Shu, an agricultural book of China written in the 1st century BC [37], the longevity of wheat and millet seeds is discussed. Their viability was maintained if they were kept as dry as possible, cool, and free from pests. It was also suggested that only large and solid ears of wheat should be chosen for sowing, and they should be dried as thoroughly as possible by the heat of the sun before storage.

Classical genetic studies were first initiated and probably the best characterized in maize (*Zea mays* L.). Maize seeds have been declared more susceptible to aging [38] if they carry homozygous alleles for either the *luteus 2* or *luteus 4* genes, although the physiological basis of this difference remains obscure. Later, two unrelated studies [39,40] demonstrated that the long-lived character of maize appeared to be dominant, although a non-cytoplasmic maternal plant influence was also identified. Thirty-seven years later, Rao et al. [41] transferred the same nuclear genotype used by Haber [40] to genetically different cytoplasmic types and concluded that cytoplasmic factors had a marked influence on seed storability. Later, single-cross hybrids were employed to investigate long- and short-lived lines of maize, which demonstrated the dominant character of the long-lived seeds [42]. After three selection cycles (based on resistance to aging using experimental procedures (42 °C and saturated humidity)), a reduction in sensitivity to aging was reported, which suggested that genetic improvement for storability is achievable [43].

The longevity of spring and winter wheat remains controversial. For example, according to Van der Mey et al. [44], winter wheat stores better than spring wheat over periods of 15–20 years at 5 °C. In contrast, Arif et al. [45] did not find any difference in longevity between spring and winter wheat after experimental aging. Furthermore, [46] reported no association between grain color and longevity in wheat.

In legumes, hard seeds within a particular seed lot retain viability for longer storage periods than their softer companions [47]. Seed color and coat thickness have also been reported to play role in seed longevity in chickpeas. For example, pale-seeded “Kabuli” chickpeas have also been reported to be shorter lived than “Desi” types with thicker, harder and darker coats [48]. Dark-seeded soybeans were also more resistant to storage under high humidity [49]. In *Phaseolus vulgaris*, a diallele cross-analysis was used to demonstrate that superior longevity was dominant in nature [50]. In soybean, reciprocal crosses revealed a strong maternal influence through the characteristics of the seed coat on the longevity of F_1_ seeds [51] in addition to a minor influence of the seed’s own genotype.

In a study of 55 accessions of barley stored in the Gatersleben gene bank since 1974, intraspecific variability in longevity was addressed [52]. Germination tests after 35 years of storage indicated intraspecific variability in seed longevity within barley owing to genetic determinants. A similar conclusion was drawn for *Brassica napus* L. [53] based on the results of 42 accessions. Hence, genotypic components are involved in determining seed viability. The same phenomenon of intraspecific variability towards longevity has been observed in *Sorghum bicolor* L., *Secale cereale* L., and *Linum usitatissimum* L. [54].

## 4. Genetic Studies in the 21st Century

Genetic mapping of seed longevity was initiated only in the 21st century when the molecular markers were readily available, and plant breeders started to use this new resource to decipher the genes and molecular mechanisms behind intraspecific variability in seed longevity in model, as well as crop, species [36].

### 4.1. Arabidopsis thaliana L.

The first detailed genetic analysis of longevity after natural aging was published in 2000 [55] while investigating raffinose family oligosaccharides (RFOs), including sucrose, raffinose, and stachyose. A recombinant inbred population (RIL) was developed, and subsequent quantitative trait loci (QTL) mapping revealed one major QTL for oligosaccharide OSs. Two candidate genes, *galactinol synthase* and *raffinose synthase*, were detected at the site of the major QTL. Additionally, four QTLs for storability were detected. QTLs for OS, however, lie at different positions from QTLs for storability, indicating different genetic controls of OS and longevity. Many years later, galactinol content was demonstrated to be highly correlated with seed longevity in *Arabidopsis*, and galactinol was identified as a suitable biomarker for predicting seed longevity [56].

Another detailed investigation was undertaken by Clerkx et al. [12] through the development of different RIL populations (‘*Landsberg erecta*’ × ‘*Shakdara*’) to determine variation in seed longevity after various artificial ageing protocols. One or more QTLs were identified for various traits (dormancy, speed of germination, seed sugar content, and germination) after various treatments (controlled deterioration test (CDT), H_2_O_2_ treatment, and abscisic acid), with some QTLs for different co-locating traits. Using various *Arabidopsis* mutants, the same group of scientists concluded that *abscisic acid insensitive3* (*abi3*), *abscisic acid deficient1* (*aba1*), and *aberrant test shape* (*ats*) mutants have reduced longevity [57]. Consequently, the importance of genetic background was revealed through the analysis of “double” mutants. In the same year, the role of vitamin E (tocopherols) in seed longevity [58] in *Arabidopsis* was demonstrated using the same mutation approach used by [57]. The authors provided evidence that tocopherols play a role in seed longevity by limiting non-enzymatic lipid oxidation during storage. In contrast, Gerna et al. [59] found that an *A. thaliana* T-DNA insertional line (*Atfahd* (*Arabidopsis thaliana fumarylacetoacetate hydrolase domain) 1a-1*) had extended seed longevity, whereas metabolite profiling of dry *Atfahd1a-1* seeds showed low δ-tocopherol levels. This indicates that the role of vitamin E in extending seed longevity is complex. In another investigation using mutant technology in *Arabidopsis,* employing genotypes with altered expression of protein L-isoaspartyl methyltransferase (PIMT1), it was concluded that the PIMT repair enzyme system contributes to seed longevity in concert with other anti-aging pathways to improve seed longevity and vigor [60].

Subsequent experiments, based on RNA interference against the three seed-expressed dehydrins, viz. LEA14, XERO1, and RAB18 (responsive to abscisic acid 18), revealed that at least one of the three seed-specific dehydrins plays a role during long-term cold storage at low moisture content [61]. Further developments in our understanding of the genetic basis of longevity after both long-term storage and artificial aging (AA) in *Arabidopsis* was made one year later with the simultaneous analysis of six RIL populations where five loci were discovered (*germination ability after storage 1* (*GAAS1*) to *GAAS5*). Although *GAAS* loci co-located with dormancy loci (*delay of germination* (*DOG*)), a negative correlation between longevity and dormancy was observed [62].

Through mutant analysis and a forward genetics approach in a subsequent study, under both natural and accelerated aging treatments, a RING-type zinc finger putative ubiquitin ligase was identified as imparting a long life to *Arabidopsis* seeds [63] by enhancing responses to gibberellins (GAs). This gene was named *RSL1* (from the ring finger of Seed Longevity1). The role of GAs was further clarified in a similar study using the same mutant approach and it was concluded that GAs may act in seed coat reinforcement [64].

To understand the influence of selective environmental influences on seed longevity, 12 *Arabidopsis* mutants varying in different seed attributes were harnessed [65] and temperature was demonstrated to play a dominant role in seed longevity after AA treatments, whereas light affected plant traits more. Furthermore, individual genotypes responded differently to different environmental conditions. For example, low temperature increased longevity and decreased dormancy in two mutants. Likewise, low light intensity also increased and decreased dormancy and longevity, respectively, in two mutants, demonstrating that different molecular pathways are involved in longevity and dormancy [62].

Further developments to understand seed longevity in *Arabidopsis* after CDT were made when tonoplast intrinsic proteins (TIP3;1 and TIP3;2) were studied in *abi3-6* mutants, where TIP2 transcript and protein levels were significantly reduced in the mutants. It was concluded that TIP3s may help extend seed longevity under the expressional control of ABI3 during seed maturation. Thus, TIP3s are members of the ABI3-mediated seed longevity pathway, together with small HSPs and late embryogenesis abundant (LEA) proteins [66]. Simultaneously, a proteomics approach was employed to obtain a deeper mechanistic view of longevity in *Arabidopsis.* Aged dry seeds and after-ripened seed proteomes were markedly dissimilar and showed that antioxidant systems, including vitamin E, are essential for seed longevity. The abundance of seed storage proteins (SSPs) also indicated that they act as buffering agents to protect seeds against oxidative stress during storage [67].

A role for genome integrity has been critically addressed in seed longevity in *Arabidopsis* [68]. Using mutant resources of *Arabidopsis*, seeds of two mutants (*atm* and *atr)* were found to be highly resistant to aging. Therefore, *ATAXIA TELANGIECTASIA MUTATED* (*ATM*) and *RAD3-RELATED* (*ATR*) are important determinants of seed viability. Taken together, the physiological functions of sensor kinases, including *ATM* and *ATR*, in linking genome integrity to germination, which influence seed quality, are crucial for plant survival in the natural environment and sustainable crop production. The same year also witnessed the use of ectopic expression methodology to understand the role of 1-cys peroxiredoxin (1-Cys Prx aka *PER1*) in seed longevity after experimental ageing [69]. Seed-specific *PER1* protein from the sacred lotus (*Nelumbo nucifera* Gaertn.) NnPER1 is ectopically expressed in *Arabidopsis* and causes enhanced germination after aging. The main reason for this enhanced germination was the significantly lower levels of ROS release and lipid peroxidation.

The year 2017 saw the use of RNA sequencing technology (RNA-seq) to address seed longevity in *Arabidopsis* during priming after CDT [70]. In total, three QTLs were detected in 279 RILs derived from cross “Est-1 ×” Col-0”. RNA-Seq analyses revealed that brassinosteroid (BR) biosynthesis/signaling and cell wall modification genes were differentially expressed in primed seeds with poor longevity. Positive BR signaling (to some extent) is thought to be the probable cause of increased permeability of the seed coat, resulting in poor seed longevity. The role of SSPs in longevity and seed germination has been investigated [71] and this study demonstrated that aspartic protease ASPG1 (ASPARTIC PROTEASE IN GUARD CELL 1) is also key factor in seed viability of *Arabidopsis* seeds. Using *Arabidopsis* mutants, they found that the SSPs during germination in *aspg1-1* mutants were very impaired in both naturally aged and artificially aged (after CDT) seeds, which led them to conclude that ASPG1 is an important player in seed longevity, dormancy, and germination, and acts via SSPs degradation and regulation of GA signaling.

Cytoplasmic genomes are an additional source of natural variation in seed longevity [72]. Cytoplasmic genomes include chloroplast and mitochondrial genomes, which differ from the nuclear genome [73]. A role for cytoplasmic genomes was identified when seed dormancy, longevity, and germination performance with natural and new genomic compositions were investigated to gain a deeper understanding of all three traits in *Arabidopsis*. Surprisingly, all traits were modified by cytonuclear reshuffling, with certain combinations providing favorable effects of novel cytonuclear combinations on longevity and other traits demonstrating the existence of suboptimal genetic combinations in natural populations for these traits. Furthermore, certain combinations exhibited a positive influence on longevity compared to natural combinations. In addition, to shed further light on the role of oxidative stress in seed longevity after subjecting seeds to CDT, another study [74] found that *Arabidopsis* seeds lacking functional NADP-MALIC ENZYME 1 (NADP-ME1) have reduced seed viability relative to the wild type. Furthermore, NADP-ME1 loss-of-function mutant seeds exhibited higher levels of protein carbonylation. NADP-ME1 catalyzes the oxidative decarboxylation of malate to pyruvate with the simultaneous production of CO_2_ and NADPH, whose expression is increased in imbibed aged seeds compared with non-aged seeds. Its activity during testa rupture promotes the normal germination of aged seeds. Hence, it was concluded that NADP-ME1 activity is required to protect seeds against oxidation during dry seed storage.

In addition to developments in genetics and genomics, the role of proteins in seed longevity after CDT has also been addressed. For example, [75] investigated the role of retromers (multi-protein complexes) in seed longevity using comparative proteomic and metabolomic analyses in the wild-type and the null-retromer mutant vacuolar protein sorting 29 (*vps29) Arabidopsis* mutant. Major changes were observed in the retromer mutant with respect to SSPs and synthesis of lipid reserves. These changes negatively altered vigor and longevity. It was concluded that retromers stimulate energy metabolism, including cell wall biogenesis, underlining the importance of retromer function in seed biology.

In 2020, natural variations of 270 ecotypes of *Arabidopsis* were used to map seed longevity loci by employing a genome-wide analysis, and several multiple genomic regions associated with variation in seed longevity were detected after subjecting seeds to various experimental aging conditions, as well as natural aging [76]. Furthermore, reverse genetics identified seven positive (*PSAD1*, *SSLEA*, *SSTPR*, *DHAR1*, *CYP86A8*, *MYB47*, and *SPCH*) and five negative (*RBOHD*, *RBOHE*, *RBOHF*, *KNAT7*, and *SEP3*) seed longevity genes. In addition, the protective role of the seed coat during seed aging was strengthened by cytochrome P-450 hydroxylase, CYP86A8, and transcription factors, *MYB47*, *KNAT7* and SEP3. The same group also reported the up-regulation of several peroxidase genes in an *Arabidopsis* mutant (*cog1-2D*) [77] with enhanced seed longevity. Furthermore, they found that seeds of double (*prx2 prx25)* and triple (*prx2 prx25 prx71)* mutants possessed reduced longevity because of low seed coat permeability. Altered polyphenolics were concluded to be the likely reason for low permeability (and hence reduced seed longevity).

More advancements took place in 2021, when it was demonstrated that the *AtHB25* transcription factor regulates seed permeability and longevity in naturally aged seeds as well as in seeds subjected to CDT by increasing the accumulation of lipid polyesters in the seed coat [78]. They further demonstrated that the AtHB25 binding target is the lipid polyester biosynthetic gene long-chain acyl-CoA synthetase 2 (*LACS2*). Its importance in seed longevity was also demonstrated by transferring *LACS2* into wheat and tomato, thus identifying AtHBD25 as a trans-species regulator of seed longevity. Finally, *AtFAHD1* [59] has also been nominated as an important agent influencing seed longevity in *Arabidopsis.*

### 4.2. Rice (Oryza sativa L.)

In crop plants, seed longevity studies were first initiated in rice in 2002 when three QTLs in 98 backcross inbred lines (BILs) on chromosomes 2 (*qLG-2*), 4 (*qLG-4*) and 9 (*qLG-9*) were identified after accelerated aging treatment [79]. Three years later, another 12 QTLs on chromosome 7 (one region) and chromosome 9 (two regions) in a set of 191 RILs after normal and experimental ageing treatments were reported [80]. Another three QTLs for storability in 127 doubled haploid (DH) lines on chromosomes 9 (*qLS-9*), 11 (*qLS-11*)*,* and 12 (*qLS-12*) following AA treatments were subsequently identified the following year [81].

Using chromosome segment substitution lines (CSSLs), the role of hull, seed coat, and embryo on the effect of rice storability QTLs (*qLG-9*, *qLG-2*, and *qLG-4* QTLs) reported by [79] was investigated [82] after being subjected to CDT. No maternal effects of the hull or seed coat were detected in the case of *qLG-9.* Embryonic and/or endospermic factors were concluded to influence longevity. Consistent with previous reports, another three rice storability QTLs were detected on chromosomes 1, 3, and 9 [83] using artificial aging protocols. Thus, chromosome 9 was thought to be targeted for longevity gene cloning in rice, which was achieved in 2015 when the chromosomal location of *qLG-9* was fine-mapped in a 30 kb interval (defined by two markers, *CAPSb* and *CHPa12*) [84]. Furthermore, two genes ((encoding trehalose-6-phosphate phosphatase (TPP) (*Os09g0369400*) and an unknown protein (*Os09g0369500*)) were annotated in this region.

In 2008–2009, the rice community replicated research in *Arabidopsis* when the potential of mutants was used to understand seed longevity. It was demonstrated that a rice aldehyde dehydrogenase (*OsALDH7*) plays an important role in maintaining seed viability after accelerated ageing treatments by detoxifying the aldehydes generated by lipid peroxidation [85]. Later, six longevity QTLs (with three QTLs (*qMT-SGC5.1, qMT-SGC7.2*, and *qMT-SGC9.1* on chromosomes 5, 7, and 9, respectively, in one RIL population) and three QTLs (*qDT-SGC2.1, qDT-SGC3.1*, and *qDT-SGC9.1* on chromosomes 2, 3, and 9 in another RIL population)) were detected after various storage periods [86]. Similar to previous studies, chromosome 9 was identified in both RIL populations. In another study, six QTLs, *qSS-2*, *qSS-3*, *qSS-4*, *qSS-6*, *qSS-9*, and *qSS-11*, on chromosomes 2, 3, 4, 6, 9, and 11, respectively, for rice storability were identified in a set of 182 BILs after 32 and 48 months of storage at 40–60% relative humidity, where *qSS-9* was the most stable [87] as it was detected in seeds from all environments and storage times, further supporting the idea of map-based cloning of *qSS-9* to gain an understanding of seed storability in rice and possibilities for its improvement. However, before the storability QTL on chromosome 9, *Os03g0700400* was identified as a candidate gene for a seed lipoxygenase (*sLOX3*) QTL on chromosome 3, after application of a map-based cloning strategy. It was determined that *sLOX3* negatively influences seed longevity, probably by facilitating the colonization of some seed pathogens [88,89]. Later, another member of the LOX gene family, *OsLOX2*, was found to act like *sLOX3* in rice seed longevity after accelerated aging [90].

Another related investigation used a set of 85 BILs to locate seed storability QTLs in rice under natural storage conditions and after AA [91]. The authors reported a total of 13 QTLs for seed storability on chromosomes 1, 2, 3, 4, 5, 7, 11, and 12, where two QTLs on chromosome 2 (*qSSh-2-1* and *qSSh-2-2*) were repeatedly detected in both treatment conditions, whereas the remaining four (*qSSh-4*, *qSSs-5-1*, *qSSs-5-2*, and *qSSh-12*) and seven QTLs (*qSSh-1*, *qSSh-3-1*, *qSSh-3-2*, *qSSh-3-3*, *qSSh-7-1*, *qSSh-7-2*, and *qSSh-11*) were detected only once in the natural and artificial aging treatments, respectively. The existence of several QTLs (*qSSh-1*, *qSSh3-1*, *qSSh-3-2*, *qSSh-3-3*, *qSSh-4*, *qSSh-7-1*, *qSSh-7-2*, and *qSSh-11*) was confirmed using CSSLs.

With the development of more sophisticated technologies to develop rice mutants in 2015, it was demonstrated that the *PIMT* gene in rice (*OsPIMT1*) increases seed longevity during AA, probably via its involvement in the repair of detrimental isoAsp-containing proteins that accumulate in acceleratory aged embryos [92].

Pioneer association mapping (AM), also known as genome-wide association study (GWAS), was initiated in 140 rice genotypes [93], and 10 associated markers were detected for longevity after experimental aging, on chromosome 1 *(RM283*, *RM81*, and *RM495*), chromosome 2 (*RM174*), chromosome 4 (*RM124*), chromosome 7 (*RM348*, *RM248)*, chromosome 8 (*RM433*, 337), and chromosome 9 (*RM160*). Additionally, a clarification of the role of tocopherols in seed longevity after aging at high temperatures in rice [94] was made when it was concluded that the specific ratio of tocopherol homologues is more important than the total tocopherol content in the seed longevity mechanism. Two years later, it was discovered that high γ-tocotrienol levels enhanced seed longevity, whereas a high proportion of β-tocopherol relative to δ-tocopherol reduced seed longevity [95]. A further development in understanding rice seed storability was made using transgenics when it was demonstrated that aldo-ketoreductases (*AKR1*) enhanced seed longevity by detoxifying toxic compounds and glycation products [96]. In 2019, the role of microRNAs (miRNAs) in seed longevity after AA treatment was addressed [97]. Up-regulation of *osa-miR164c* and down-regulation of *osa-miR168a* were observed in aged seeds. Concomitant changes in the cytomembrane permeability of seeds and the expression of *osa-miR164c* target genes (*OsPM27* and *OsPSK5*) and *osa-miR168a* target genes (*OsAGO1* and *OsPTR2*) under aging conditions coincided with changes in seed vigor induced by *osa-miR164c* and *osa-miR168a*. Hence, miRNAs can be targeted for future research and improvement in longevity.

Another genetic study mapped novel loci linked to seed longevity in rice using 172 RILs after natural aging. It uncovered two QTLs, *qSL-2* and *qSL-8*, on chromosomes 2 and 8, respectively, where the latter was regarded as a novel QTL for longevity [98]. The study of metabolites sheds further light on seed longevity in rice when it was discovered that amino acid-related and sugar-related metabolites were active in seeds with poor storability. However, raffinose levels were lower in seeds with better storability [99], indicating that raffinose can be used as a marker for seed longevity. More loci for seed longevity have been detected on chromosomes 1, 3, 4, 9, and 11 through AM [100]. Chromosome 2 was demonstrated to be resident of a natural longevity QTL in a tropical japonica rice in a F_3_ bi-parental population with 45 annotated genes potentially relevant to seed longevity located in that area [101]. AM was further convened in 456 rice core collections and produced nine QTLs (*SS1-1*, *qSS1-2*, *qSS2-1*, *qSS3-1*, *qSS5-1*, *qSS5-2*, *qSS7-1*, *qSS8-1*, and *qSS11-1*) [102] after accelerated aging treatments. They also confirmed that *qSS1-2* and *qSS8-1* colocalized with *the qSS1*/*OsGH3-2* and *OsPIMT1* loci. Finally, bulked segregant analysis of two BILs through whole-genome sequencing (BSA-seq) was used to locate potential longevity QTLs when subjected to AA treatments [103]. Two main genomic regions containing 18,550,000–20,870,000 bp on chromosome 4 and 7,860,000–9,780,000 bp on chromosome 9 were identified, and 448 annotated genes were predicted.

### 4.3. Barley (Hordeum vulgare L.)

Following the successful application of genetic studies in rice, investigations have started in other crops, including barley. Three DH mapping populations were employed by [52] viz. ‘Steptoe’ × ‘Morex’ (S × M) population (94 DHLs), the OWB population (94 DHLs) and the W766 population (100 DHLs), which were subject to both AA and CDT. For “S × M”, a highly significant QTL was located on chromosome 5HL. In the case of OWB, three QTLs were detected on chromosomes 2H, 5H, and 7H. Finally, in W766, a single QTL on chromosome 7H was identified. The candidate genes on chromosome 7H were suggested to be the “nud” gene that segregates for the character hulled/naked caryopsis [104]. The hulled trait contributed to superior longevity. On chromosome 2HL, *Zeo1* (responsible for small plant stature with compact spikes, long awns, and reduced fertility) [104] was found to be responsible for differences in seed longevity. On chromosome 5H, one of the candidate genes was *Aleurain* (*Ale*) which is a barley vacuolar thiol protease whose expression is regulated by the plant hormones gibberellic acid and abscisic acid. On chromosome 2H, dehydration responsive element binding protein (DREB) was reported as a candidate gene, which, together with the ethylene-responsive element (ERE) binding factors, belong to the APETALA2/ethylene-responsive element-binding protein family that play an important role in the regulation of abiotic and biotic stress responses, respectively. DREB expression is activated by drought, cold or ethylene [105]. On chromosome 5H, other genes, including thaumatin-like proteins such as *Barperm1*, have been proposed as a candidate gene [106]. AM analysis was used by [107] and they detected 55 loci for normal seedling appearance and 36 loci for total germination in a set of 175 genotypes (122 landraces and 53 cultivars), which were mainly concentrated on chromosomes 2H, 5H, and 7H, thus confirming previous results [52]. The most important candidate genes on chromosome 2H included the DREB protein, stem rust resistance protein RPG1, putative gag-pol polyprotein, RNaseH (Ty1/Copia family), and ABC transporter C family member 10, whereas the candidate genes on chromosome 5H included the WD40-like beta propeller repeat family protein, thaumatin-like protein TLP5, *Barperm1,* heat shock cognate 70 kDa protein 2, and APETALA2-like protein. Enoyl-ACP reductase, ethylene-responsive element-binding factor 1 (EREBP-1), and sucrose synthase have been reported at the site of chromosome 7H loci.

More refined effort towards genetic understanding of seed longevity in barley was attempted by [108] through a combination of quantitative genetics and “omics” approaches in near isogenic lines (NILs) derived from crosses between the spring barley landraces “L94” from Ethiopia and “Cebada Capa” from Argentina. RNA-seq and total seed proteomic profiling identified the UDP-glycosyltransferase MLOC_11661.1 as a candidate gene for the quantitative trait locus on chromosome 2H, and the NADP-dependent malic enzyme (NADP-ME) MLOC_35785.1 as possible downstream target gene. This finding was validated using ectopic expression of the aforementioned genes in *Arabidopsis* under the control of constitutive promoters. Both NADP-ME MLOC_35785.1 and UDP-glycosyltransferase MLOC_11661.1 were able to restore the nadp-me1 seed longevity phenotype.

Further developments revealed the association of miRNAs with seed aging in barley in an investigation that involved distinctive seed lots that belonged to a single genotype (cv. ‘Damazy’) which varied in viability after over 45 years of storage in a dry state [109]. The dry seeds carried 142 miRNAs, 81 of which were novel. Four conserved miRNA families (miR159, miR156, miR166, and miR168) were highly expressed. Surprisingly, almost all miRNA levels were similar in both highly viable and low viability seed lots, providing evidence that miRNAs remained unaffected during long-term storage. The authors also detected a novel miRNA, viz. hvu-new41, which could be used as an indirect marker to determine seed viability in barley.

### 4.4. Wheat (Triticum aestivum L.)

The first report addressing the genetic basis of longevity in bread wheat appeared in 2010 reporting a pilot investigation of seed longevity traits in wheat/*Aegilops tauschii* introgression lines following AA [110]. Overall, five QTLs for seed longevity were reported on *Aegilops* chromosomes 1D and 5D, indicating the existence of genetic variability in seed longevity in wheat. This was followed by a detailed investigation in 2012 of classical linkage mapping (performed on 114 RILs of the “International Triticeae Mapping Initiative” mapping population (ITMI/MP)) and GWAS was performed on 96 winter wheat accessions [111]. In ITMI/MP, longevity loci were detected on chromosomes 1A, 1D, 2A, 2D, 3B, 3D, 6B, and 7A using both CDT and AA. Interestingly, the loci detected after CDT and AA treatment were distinct. The region detected on chromosome 2A also harbors many pathogen defense response genes. In particular, the QTL is flanked by *Per2* (peroxidase) and *Wip* (wound-induced protein) genes [112], whereas the 3B locus is implicated in yield-related traits, including grains per ear and thousand grain weight [113]. The chromosome 1A locus has been mapped close to a QTL for spike compactness [114]. Longevity loci in ITMI/MP were reported for relative germination rates after the ageing treatments [111]. The authors of [115], using the unpublished data of [111] and some newly generated data in the same ITMI/MP, revealed some new loci, as well as those already reported in [111], for absolute germination before and after aging treatments. Seed longevity loci were located on chromosomes 1A, 1B, 2B, 2D, and 3D (two similar and one distinct loci) and 4A, 5D, 6B, and 7B (two similar loci), indicating the quantitative nature of longevity related traits in wheat.

**Figure 1:**
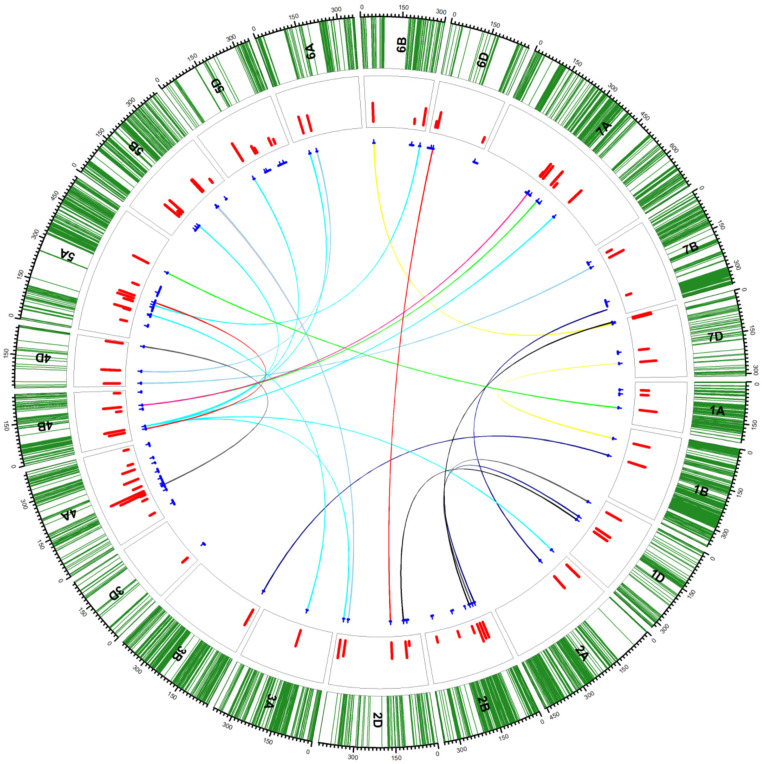
Circos diagram showing the presence of additive (unconnected blue lines in the inner circle) and epistatic (connected blue lines in inner circle) QTLs. Green lines in the outer track indicate the SNP positions on each chromosome; red bars in the second circle indicate the LOD values of QTLs. The blue lines under the track circle indicate the confidence interval of QTLs with small vertical lines point to the peak position of QTL. The colored lines linked different biallelic epistasic QTLs (yellow, pink, sky blue, navy blue, aqua, black, deep pink and red indicate traits related to seed longevity in either control or after various treatments and green and grey and grey indicate dormancy related loci. The figure is reproduced from [117].

The studies of [111,115] were performed with a basic genetic map consisting of 942 loci (mainly “single sequence repeats” and some “restriction fragment length polymorphism” markers) with limited coverage. Recently, a substantially more saturated map of ITMI/MP comprising 7584 single-nucleotide polymorphism (SNP) markers has been published [116]. This new information was utilized [117] to identify novel loci related to longevity using the phenotypic data of [111] and [115]. Overall, 31 loci were reported on chromosomes 1A, 1B, 3D, 4A, 4B, 5A, 5B, 5D, 6B, 6D, 7A and 7B for longevity. In addition, 14 other loci were detected for dormancy-related traits on chromosomes 1A, 2D, 4A, 4B, 5A, 6D, and 7D (Figure 1).

In addition to additive QTLs, epistatic QTL networks of seed longevity loci were reported for the first time in ITMI/MP [117], which was only made possible after the arrival of more sophisticated statistical tools [118] to elucidate genetic networks of the loci responsible for agronomically important and complex traits in polyploid species. The most important but novel candidate genes associated with seed longevity-related traits were detected by the authors of [117], including *ankyrin (ANK)-3-like isoform X1* and *60S ribosomal protein L10a*, *pentatricopeptide repeat-containing protein*, *geraniol 8-hydroxylase-like*, *ETHYLENE-INSENSITIVE 2-like* (*EIN2*) and *RDM16-like isoform X2*, and *carotenoid 9,10(9′,10′)-cleavage dioxygenase-like isoform X1* and *aspartyl protease family protein 2-like*. ANK proteins play a prominent role in plant immunity, development, and growth by being involved in protein–protein interactions [119], whereas members of the pentatricopeptide repeat (PPR) protein family are sequence-specific RNA-binding proteins that are central to organelle RNA metabolism [120]. Geraniol 8-hydroxylase-like proteins are associated with *MAP kinase* signaling pathways [121]. *EIN2* governs stable miRNA164 expression during aging [122]. Likewise, *carotenoid cleavage deoxygenases* that cleave carotenoids and apocarotenoids are produced by their actions, which play various roles in the growth and development of plants [123]. Members of the aspartyl protease family regulate plant defense responses via different means [124]. Some of these genes have recently been reported to impart *Fusarium* head blight resistance [125].

Thirty-eight diversity array technology (DArT) markers out of a total of eight hundred and forty were reported to be associated with seed longevity-related traits in the GWAS analyses [111], and a number of candidate genes have been proposed. This population was later covered by a substantially higher number of SNP markers. Overall, 11,139 SNPs were mapped to this panel and the phenotypic data of [111] were reanalyzed in [7]. A more refined analysis uncovered 16 associations on chromosomes 1A, 2A (10 associations), 2D, 6A (3 associations), and 7A. The probable candidate genes reported at these loci included *rhomboid 19, disease resistance RPM1-like* and its *isoform X1*, *eukaryotic translation initiation factor 3 subunit M*, *subtilisin-like protease isoform X2*, the *ELMO CED-12 family*, and *Brahma 1*.

Another report in 2017 [45] attempted to define seed longevity loci from a collection of genebank accessions in wheat that were covered with 2134 polymorphic DArT markers. Altogether, 103, 74, and 97 loci for long-term cold storage aging, AA, and CDT, respectively, have been reported. Fifteen bins were identified as likely to contain genes that influence longevity. In addition, the loci linked with germination percentage following long-term low-temperature storage and lab treatments were largely dissimilar, probably because of the involvement of different mechanisms of deterioration [9]. This study was conducted on a collection containing both spring and winter wheat accessions [45], which could have possible implications on the detected loci, as flowering time influences the outcome of most development-related traits. In view of the above possibility, a filtered attempt was made by [7], who used a subset of spring wheat accessions reported in [45] and a new set of 9804 SNPs, combined with more stringent statistical significance. Their effort [7] revealed a total of 56 loci on chromosomes 1A, 1B (10 loci), 2A (2 loci), 2B (6 loci), 2D, 3A (2 loci), 3B, 4A (2 loci), 4B (16 loci), 5B (9 loci), 7A (3 loci), 7B (2 loci) and 7D which were confined to 20 QTLs, out of which eight QTLs were reported to be potentially novel which were located on chromosomes 2B, 3A, 4A, 5B (two QTLs), 7A, and 7D.

The data presented in [45,111] reported a large number of candidate genes, whereas the reanalysis of the data of these studies confined the probable candidate genes to 37 that can be used for future research [7]. Among them, the most important genes that have also been reported include the *stem rust resistance protein Rpg1* and *NBS*-*LRR resistance*-*like protein* [126]. Likewise, another common candidate gene was *FAR1*-*related sequence 6*-*like protein* w (also reported in [127]), which is expressed in hypocotyls, rosette and cauline leaves, inflorescence stems, and flowers, and is linked to positive regulation of the circadian rhythm and transcription. In addition, it is thought to be involved in ABA signal transduction and abiotic stress response pathways.

More reports emerged in 2018 and 2019 when loci for longevity were examined in-depth in an RIL population composed of 246 genotypes using the classical linkage mapping approach [126] and in a diverse panel of 166 varieties of wheat (144 indigenous Chinese and 22 foreign accessions) using the GWAS approach [127] where the wheat 90K iSelect SNP array (81,587 gene-associated SNPs) [128] was convened in both cases. Ninety-six QTLs were reported on all wheat chromosomes except 2B, 4D, 6D, and 7D, which were clustered into 17 QTL-rich regions on chromosomes 1AL, 2DS, 3AS (3), 3BS, 3BL (2), 3DL, 4AS, 4AL (3), 5AS, 5DS, 6BL, and 7AL, exhibiting pleiotropic effects in the former report [126]. The most promising candidate genes reported were *starch synthase 3*, *stem rust resistance protein* *Rpg1*, *NBS-LRR resistance-like protein*, *dolichyl-diphosphooligosaccharide-protein glycosyltransferase*, *glutaminyl-peptide cyclotransferase-like*, and *wheat alpha-Amy2/53.* In a latter report [127], 23 loci were reported on chromosomes 1A, 2A (2), 2B (3), 2D, 3A (2), 3B, 3D (2), 4A (3), 5A (2), 5B (3), 5D (1), and 6A, whereas the candidate genes included *FAR1-related sequence 6-like protein*, *delta-1-pyrroline-5-carboxylate synthase (P5CS)*, and *MICOS* *complex subunit mic60 protein*, which is essential for the integrity of cellular membranes and genomes [129].

The most recent report addressing seed longevity in wheat from a genetic perspective appeared in 2020 when an analysis of 150 DH lines [130] was reported after six aging treatments, in which 49 additive QTLs for seed vigor-related traits were mapped onto chromosomes 1B, 2D, 4A, 4D, 6D, and 7A and all group 3 and group 5 chromosomes. In addition, 25 pairs of epistatic QTLs were reported on all chromosomes except chromosomes 5D, 6A, and 7D. This study pioneered the mapping of epistatic loci of seed longevity in wheat. The same approach has been adopted to map epistatic loci in ITMI/MP to explain further variation in this trait [117].

### 4.5. Maize (Zea mays L.)

Natural variability of maize seed viability at the beginning of 21st century using RILs has been examined [131,132]. The investigators found that viability decreased linearly with age for most inbred lines, with a few exceptions. They also discovered that the same enduring seeds of most RILs, when sown, produced new seeds with superior viability and vigor compared to average values, suggesting natural selection for viability and vigor during storage. This prompted another study by the same group [132] and they identified genes of seed longevity in maize employing sweet corn inbred line ‘P39’ and the field corn inbred line ‘EP44’. Simple sequence repeats (SSRs) in the bulk of living and dead seeds after 20 and 22 years of storage were compared, and the differences between dead and living seeds could be explained by residual variability, spontaneous mutation, or aging. Chromosome 7 exhibited more variability than the other chromosomes. Likewise, variability was more pronounced for distal SSRs. Six known genes, including *pathogen-related protein 2*, *superoxide dismutase 4*, *catalase 3*, *opaque endosperm 2*, *metallothionein 1*, and *golden plant 2* have been reported as candidate genes for longevity. In addition, five novel candidate genes (three of which could be involved in resistance to diseases, one in detoxification of electrophilic compounds, and another in transcription regulation) were reported.

Early harvesting is preferred in maize, but it can result in poor quality and low seed vigor [133]. To tackle this, the genetics of seed vigor at different stages of maturity were determined using a set of maize RILs harvested 32, 40, and 45 days after pollination, coupled with a genetic linkage map covering a distance of 2438.2 cM through 217 SSRs. In total, there were sixteen different QTLs for seed vigor at three sampling times, five for germination energy (number of seedlings on day 3 divided by total seeds sown), three for germination percentage, four for germination index, and four for vigor index. The four QTLs for seed vigor, which were detected at all three sampling times, were located in a comparable region on chromosome 7, confirming the findings of the authors of [132] who also reported chromosome 7 to be an important carrier of longevity genes in maize.

The first comprehensive use of SNPs for studying seed longevity also appeared in maize in 2014 [134] in two unrelated RIL populations subjected to four different treatments. Sixty-five QTLs distributed between the two populations were identified, from which sixty-one were integrated into eighteen meta-QTLs (mQTLs). Finally, twenty-three candidate genes coincided with thirteen mQTLs. Eight of these genes were associated with the glycolytic pathway. The functions of two of the candidate genes were associated with stress responses, including a *stress-responsive glyoxalase family* gene and an *aldehyde dehydrogenase* gene. The functions of the remaining six candidate genes were associated with energy processes, including an *ATP synthase F1 subunit alpha gene*, *glyceraldehyde 3-P dehydrogenase* gene, *V-type (H+)-ATPase domain* gene, *phosphoglucomutase* gene, *3-phosphoglycerate kinase* gene, and *isocitrate lyase* gene. Eight candidate genes were functionally associated with protein metabolism. Four candidate genes had functions related to general protein metabolism or translation, including an *elongation factor 1-g2* gene, an *elongation factor 1B-g* gene, a *calreticulin 1* gene, and an *Asp aminotransferase* gene. The other four candidate genes encoded HSPs, including a hypothetical *ACD ScHsp26-like* gene, *HSP16.9*, *HSP17.2*, and *hsp20/alpha crystallin* family protein gene. In addition, five candidate genes were associated with protein modification and signal transduction. These included a *ubiquitin E2* gene, *calcium-dependent protein kinase,* a gene containing a predicted *RING-finger domain*, a *CAAX prenyl protease 1* gene, and a *cucumisin-like serine protease*. Two other candidate genes had functions related to cell growth and division, including a predicted *cyclin-dependent kinase A* gene and an *MEK homolog1* gene.

The RIL population used in [133] and an immortalized F_2_ (IF_2_) population were convened in 2015-16 to examine genetic differences between homo-and heterozygous maize lines after AA and subsequent evaluation in field experiments [135]. Twenty-eight and twenty-one QTLs were detected in the IF_2_ and RIL populations, respectively, with only one QTL (*qGP5*) common between both. In the IF_2_ population, a QTL (*qGI10b*) was detected on chromosome 10 in the same region as that reported in the same population [133], which demonstrated that this locus corresponds to major genes associated with seed germination or seed aging. Likewise, a QTL on chromosome 3 (*qGE3a*) in the RIL population in this study was the same QTL previously reported [133]. In addition, candidate genes for QTLs *qVI4b* and *qGE3a* detected in the RIL population were suggested to be *ZmLOX1* and *ZmPLD1.*

Another report on QTL mapping for seed longevity in maize after AA appeared in 2018 in an F_2:3_ population and RIL population [136]. The authors reported 13 QTLs on chromosomes 1, 3, 4, 5, and 7, where 2–4 QTLs were co-located in one region. In each region, three to eight previously identified aging-related QTLs were located, confirming the importance of these regions in controlling seed longevity in different maize populations. The parents (I178 and X178) of the same population used in this study [136] were also subjected to transcriptome sequencing before and after five days of AA treatment [137], which resulted in the detection of 286 and 220 differentially expressed genes (DEGs) in I178 and X178, respectively. Of these DEGs, 98 were detected in both I178 and X178, which were enriched in Gene Ontology (GO) terms of the cellular component of the nuclear part, intracellular part, organelles, and membrane. Only 86 commonly downregulated genes were enriched in GO terms of the carbohydrate derivative catabolic process. Additionally, transcriptome analysis of alternative splicing (AS) events in I178 and X178 showed that 63.6% of transcript isoforms occurred in AS in all samples, and only 1.6% of transcript isoforms contained 169 genes that exhibited aging-specific AS after aging treatment. Combined with the reported QTL mapping result, seven DEGs exhibited AS after aging treatment, and thirteen DEGs in the mapping interval were potential candidates that were directly or indirectly related to seed longevity. The authors also reported very low expression of *ZmPIMT* and *ZmLOX11* genes in seeds, which implies that they are highly tissue-specific or even differentially expressed in mono- and dicots. In addition, several novel candidate genes have been suggested for longevity.

In 2020, the use of maize mutants provided evidence that *ZmDREB2A* regulates the longevity of maize seeds following AA by stimulating the production of raffinose while simultaneously limiting auxin-mediated cell expansion [138]. The authors detected that unaged seeds of two independent maize *DREB2A* mutant (*zmdreb2a*) lines, with decreased expression of *GRETCHEN HAGEN3.2* (*ZmGH3.2*, encoding indole-3-acetic acid (IAA) deactivating enzyme) and increased IAA in their embryos, produced longer seedling shoots and roots than the null segregant (NS) controls. In contrast, *zmdreb2a* seeds with decreased expression of *RAFFINOSE SYNTHASE* (*ZmRAFS*) and less raffinose in their embryos, exhibited decreased seed aging tolerance compared to the NS controls. When they overexpressed *ZmDREB2A* in maize protoplasts, there was an increased expression of *ZmGH3.2*, *ZmRAFS* genes and that of a *Rennila* LUCIFERASE reporter (*Rluc*) gene, which was controlled by either the *ZmGH3.2* or *ZmRAFS* promoter. They also provided evidence that *ZmDREB2A* directly binds to the DRE motif of the promoters of both *ZmGH3.2* and *ZmRAFS*.

Another attempt to map QTLs for seed longevity in maize was made in 2021 by utilizing an F_2:3_ population and a population of RILs constructed from a cross between “Dong156” and “Dong237” [139]. Genotyping was performed using SSR markers and the seeds were subjected to AA. Two consistent regions, *cQTL*-7 on chromosome 7 (also in line with [132]), and *cQTL*-10 on chromosome 10, were identified by comparing QTL analysis results of the two populations. The four SSR markers (*umc1671*, *phi328175*, *umc1648*, and *phi050*) linked to *cQTL*-7 and *cQTL*-10 could be used to select maize germplasms with a high degree of seed storability.

### 4.6. Soybean (Glycine max (L.) Merr.)

The first study to resolve the genetics of seed longevity after experimental ageing in soybean was initiated in 2008 [140] in an F_2:3_ population composed of 153 lines from a cross of ‘Birsa soya-1′ × ‘JS 71-05’. Four (*Satt538*, *Satt600*, *Satt434* and *Satt285*) independent SSR markers were significantly associated with seed longevity at a distance of 158.63 cM, 75.4 cM, 105 cM and 25.51 cM on chromosomes A2, D1b, H and J, respectively [141]. The same population was also subjected to seed coat permeability and electrolyte leaching by the same group [142], where four SSRs (*Satt434*, *Satt538*, *Satt281*, and *Satt598* on chromosomes H, A2, C2, and E, respectively) were associated with seed coat permeability. In addition, SSR *Satt281* has been linked to electrolyte leaching on chromosome C2. The SSR *Satt281* was also reported in a subsequent longevity analysis of 21 soybean genotypes [143].

To link seed coat color with longevity, Hosamani et al. used 33 genotypes of soybean that differed in storability and seed coat color with 53 SSR and 51 randomly amplified polymorphic DNA (RAPD) markers [144]. Black seed coat genotypes were more stable under both natural and AA conditions. SSRs grouped the genotypes into two major clusters, representing black- and yellow-seeded genotypes. SSR markers *Satt371*, *Satt453*, and *Satt618* were identified as candidate markers for linkage with seed storability and testa color. In the same year, a QTL mapping study of soybean for seed longevity in an F_2:3_ population derived from a soybean line (MJ0004-6; characterized by poor longevity) and a landrace cultivar from Myanmar (R18500; characterized by superior longevity) was published [145]. The F_2:4_ seeds after AA, as well as under ambient conditions, revealed 13 markers from six linkage groups (C1, D2, E, F, J, and L) in association with seed storability. QTL mapping detected three QTLs on linkage groups C1, F, and L, and the SSRs involved were *Satt476* and *Satt399*, *Satt269*, *Satt423*, *Satt523*, and *Satt143*, respectively.

Another investigation to elucidate the genetic mechanism behind seed longevity was undertaken during detailed physiological and molecular characterization of late seed maturation [146]. A two-fold increase in longevity was observed by the time seeds were dry. This increase in longevity was linked to the expression of genes encoding protective chaperones, including HSPs, and the repression of nuclear and chloroplast genes involved in a range of chloroplast activities, including photosynthesis. An increase in the RFO/sucrose ratio, together with changes in RFO metabolic genes, was also associated with longevity. Twenty-seven transcription factors were identified, whose expression profiles were highly correlated with longevity, including homologues of *ERF110*, *HSF6AB*, *NFXL1*, and members of the *DREB2* family, as well as transcription factors associated with auxin and developmental cell fate during flowering, organ growth, and differentiation. Transcriptional transition occurred concomitantly with seed chlorophyll loss and detachment from the mother plant, which was enriched with AP2/EREBP and WRKY transcription factors and genes associated with growth, germination, and post-transcriptional processes that prepared the seed for the dry quiescent state and subsequent germination.

A comprehensive investigation to identify QTLs employing high-density genetic linkage maps of soybean for seed longevity in two RIL populations derived from “Zhengyanghuangdou” × “Meng 8206” and “Linhefenqingdou” × “Meng 8206” using both natural and AA protocols identified 34 QTLs on 11 chromosomes, all of which were novel [147]. Twenty-one of these QTLs were clustered in five QTL-rich regions on four chromosomes: Chr3, Chr5, Chr17, and Chr18. In addition, “QTL hotspot A” on Chr17 and “QTL hotspot B” on Chr5 carried seven and six QTLs, respectively. Hence, stable genomic regions governing the inheritance of seed storability in soybeans have been reported.

Biochemical and molecular analyses of the parents exhibiting black (Birsa soya-1) and yellow seed coat color (EC 241780) and the 11 F_3_ progenies of the cross exhibiting brown, yellow, and black seed coat color have been undertaken [148] to understand the physicochemical attributes related to soybean seed longevity. Vitamin E, lignin, calcium content, and antioxidant enzyme activity were higher in black and brown seed coat color progenies, whereas lower lipid peroxidation rates were recorded in black and brown seed coat color parents and progenies with better seed longevity. The SSR primers *Satt162, Satt523*, and *Satt453*, which are linked to seed coat color and seed permeability, exhibited a specific-size allelic fragment in soybean genotypes and crosses with better seed longevity, confirming the results of a previous report [142].

The latest report on seed longevity in soybean appeared in 2021, where the investigators analyzed the QTLs for seed longevity in an RIL population under −20 °C conservation and AA conditions [149]. Two major QTLs and eight QTL hotspots localized on chromosomes 3, 6, 9, 11, 15, 16, 17, and 19 were detected for a variety of seed vigor-related traits across the two treatments. No common QTLs were detected in the RIL populations. Furthermore, 15 promising candidate genes were reported that could possibly determine seed vigor in soybeans, which would help to explore the mechanisms responsible for maintaining high seed vigor.

### 4.7. Lettuce (Lactuca sativa L.)

Seed longevity has been thoroughly explored at the genetic level in one report [150]. QTL mapping for seed longevity after conventional storage (30% RH and various temperatures for various durations) and CDT on RILs produced from a cross between “*Lactuca sativa* cv. Salinas” × “*Lactuca serriola* accession UC96US23” identified multiple QTLs under CD and conventional storage conditions. However, they did not find a correlation between the results of CD and conventional storage, which led to the conclusion that CD conditions are not predictive of aging in conventional storage environments. QTLs for seed longevity were reported on chromosomes 1, 2, 3, 4, 6, 7, and 8 after various treatments, where chromosomes 1 and 4 were involved in multi-treatment QTLs. Four genes (*AtHSF4*, *AtIRX14*, *AtMAT3*, and *AtUBC16*) were suggested as candidate genes on chromosome 4, including *heat shock transcription factor 4, phosphoglycerate/biphosphoglycerate mutase* protein family, *CC-NBS-LRR* and *TIS-NBS-LRR* class disease resistance protein, *glycosyl transferase* family 43, *methionine adenosyltransferase 2*, *ubiquitin-conjugating enzyme 16*, and *pfkB-type carohydrate kinase* protein family. The other locus on chromosome 4 carried *AtPAP2* as a candidate gene, which produces phytochrome-associated protein 2 (AUX/IAA family). There were other possible candidate genes, including *AMP-dependent synthetase* and *ligase,* a *zinc finger* (*GATA* type) family protein, an *ABC transporter* family protein, and *LOX*. The two regions on chromosome 1 were reported to carry approximately nine candidate genes, viz. *AtCPN60A*, *AtCKS1*, *AtMTN2*, *AtABA2*, *AtCYSA*, *AtEIN2*, *AtBGL2*, *RPT1A*, and *AtHSD5*, which include *RuBisCO subunit binding protein*, *cyclin-dependent kinase*, *methyladenosine nucleosidase*, *ABA2*, *cysteine-type endopeptiase inhibitor*, *EIN2*, *glucosidase*, *26S proteasome AAA-ATPase subunit RPT1a*, and *hydroxysteroid dehydrogenase 5*.

### 4.8. Tobacco (Nicotiana tabacum L.)

One of the first systematic studies to approach seed longevity loci under ambient conditions in tobacco was initiated on a collection of 122 RILs derived from a cross between the cultivars “Florida 301” and “Hicks” [151]. They were able to detect four genomic regions located in four different linkage groups (LGs): 6, 7, 8/18 and 23. The loci on LGs 6 and 7 carried loci specifically detected under treated and controlled conditions, respectively, whereas LGs 8/18 and 23 carried loci detected under both controlled and treated conditions at almost the same locations. The LG 8/18 locus was reported in the same region where a disease resistance locus against black shank disease caused by *Phytophthora nicotianae* was detected in a previous study [152]. A subsequent study examined 118 DHLs developed from a cross between cultivars, “Beinhart-1000” and “Hicks” [153] where another 24 loci were detected on 11 linkage groups. Additional variation was captured by performing epistasis analysis, which resulted in the detection of five pairs (four in the control group after treatment) of epistasis QTLs. Thus, epistatic QTL exploration for seed longevity has become an active topic, and it is expected that with the development of more sophisticated QTL analysis tools, more epistatic QTLs will be identified.

### 4.9. Tomato (Solanum lycoperscum L.)

To address the problem of reduced seed longevity after AA in tomato, QTL mapping in 50 RILs of tomato (from a cross of “*Solanum lycopersicum* cv Moneymaker” × *Solanum pimpinellifolium*) was performed [56]. Two QTLs were detected on chromosome 2, and one on chromosome 6. Two additional QTLs were detected for galactinol content on chromosomes 2 and 4. One of the QTLs for longevity and galactinol content on chromosome 2A falls into the same interval as the *galactinol synthase* gene (*Solyc02g084980.2.1*), which is a key enzyme of the RFO pathway. A reverse genetics approach using T-DNA knockout lines in genes encoding enzymes of the RFO pathway (*galactinol synthase 1, galactinol synthase 2, raffinose synthase, stachyose synthase, and alpha-galactosidase*) and overexpression of the cucumber *galactinol synthase 2* gene in *Arabidopsis* was undertaken. The analysis demonstrated that the galactinol synthase 2 mutant and galactinol synthase 1 galactinol synthase 2 double mutant contained the lowest seed galactinol content, which coincided with lower seed longevity. It was thus concluded that galactinol content of mature dry seeds can be used as a biomarker for seed longevity in Brassicaceae and tomatoes.

Seed priming is a way to achieve synchronized and rapid germination in tomatoes. However, it comes at the cost of reduced longevity. This can be ameliorated by heat shock to primed seeds. The genetic determinants of this improvement in longevity were investigated using primed and dried tomato seeds [154]. RNA-seq and subsequent transcriptome analysis of the tomato seeds after priming and after heat shock treatment post-priming demonstrated that from a total of 368 differentially expressed genes, 298 genes were up-regulated and 70 were down-regulated. An increase in messenger RNA levels of *heat shock factor-like* and *HSP-like* chaperone genes demonstrated their pivotal role in the enhancement of longevity in primed tomato seeds.

To further understand the acquisition of desiccation tolerance and seed vigor, including longevity in tomato, Bizouerne et al. [155] used temporal RNA-seq analyses of the different seed tissues during maturation. Gene networks and trait-based correlations were used to explore the transcriptome signatures associated with longevity. A total of 15,173 differentially expressed genes were detected, forming a gene network representing 21 expression modules, with three being specific to the seed coat and embryo and five to the endosperm. A gene–trait significance measure identified a common gene module between the endosperm and embryo associated with longevity, which included antioxidant and repair genes, in addition to LEA-, HSP-, and ABA-responsive genes. Longevity enhancement was correlated negatively with dormancy emancipation which was released concomitantly with the increase in longevity throughout fruit ripening, until 14 days after the red fruit stage, which also equated with an increment in SlDOG1–2 and PROCERA transcripts. The step-by-step increase in seed vigor was captured by two tissue-specific and one common gene module (between embryo and endosperm). Each module carried specific genes. For example, the common module carried genes responsible for mRNA processing, flowering time, and post-transcriptional regulation. ABI4 and CHOTTO1 were associated with seed vigor in the embryo-specific module, whereas the endosperm-specific module revealed diverse processes involved in genome stability, defense against pathogens, and ABA/GA response genes.

## 5. Future Directions

Biological and agricultural research is at a crossroads in the third decade of the 21st century, where boundaries between various disciplines are rapidly diminishing. Multiple approaches are being adopted to understand the mechanisms underlying biological processes such as seed longevity. Thus, it is imperative that researchers from different disciplines (e.g., plant breeders, seed technologists, plant physiologists, and molecular biologists) collaborate to further our understanding of the processes of seed aging. Although the quest for seed immortality is a mission impossible [156], researchers have achieved substantial success in understanding seed deterioration at the genetic, molecular, and physiological levels. However, much remains to be done.

Genetic research, in particular, involves two versatile methods: linkage analysis and association mapping to dissect quantitative traits [157] including seed longevity [111]. The former relies on trait segregation in a population derived from a bi-parental cross, whereas the latter is a population-based method that relies on the detection of linkage disequilibrium between a trait and a genetic marker in unrelated accessions [158]. Both the bi-parental population and the genetic map need to be created before phenotypic traits can be associated with the linkage groups of the plant species under consideration. For example, in the case of wheat, an SSR map was initially developed for ITMI/MP [159]. SSR maps were developed using manual pipetting in the laboratory, which took years to complete. However, with the development of genotyping technology, large-scale genotyping has been outsourced to invest the saved time in intensive phenotyping. One example is the development of DArT markers in wheat, which have been used to map important loci of dormancy and pre-harvest sprouting [160] and longevity [111]. Further developments have led to the discovery of SNPs, and readily available new populations have been genotyped using this new set of markers [116]. Hence, SNPs represent the ultimate form of molecular markers, the plant populations of any species previously mapped with primitive marker systems can be genotyped with SNPs, and the data can be used to understand the genetics of any trait.

Experimental examination of seed longevity is a time-consuming and labor-intensive process as it involves growing a population of plants for one complete cycle, harvesting the seeds and post-harvest treatments, and subsequent seed storage. The harvested/stored seeds are then treated experimentally, followed by germination tests (where germinated seeds were visually counted) to obtain an estimate of longevity in that population. With the arrival of high throughput scoring of seed germination in at least model plants (*Arabidopsis*) [161], new methods for phenotype germination on a large-scale in limited time in other plant species could soon be undertaken in future to accelerate research on the seed longevity of important plants.

Sophisticated methods of QTL detection have been developed and incorporated in various programs to facilitate breeders to capture hidden or otherwise uncaptured trait variation. Initially, in wheat, a single marker analysis was performed to map longevity loci in pioneer longevity studies [111]. Similarly, interval mapping, composite interval mapping, and inclusive composite interval mapping methods have been developed and incorporated into important QTL detection programs, including *Qgene* [162], *QTL Cartographer* [163] and *ICIM mapping* [118]. More recently, whole-genome composite interval mapping (*QTL*.*gCIMapping*) [164] has been proposed to detect small-effect QTLs. As longevity QTLs also have small effects, especially in polyploid species [3,111], *QTL*. *gCIMapping* [164] might be a useful option to explore seed longevity QTLs in future studies. All of the above-mentioned studies, with the exception of [117,130,153], have reported additive QTLs. However, further variation can be detected by exploiting epistatic interactions among loci to explain the genetic determinants of seed longevity. The detection of epistatic interactions is possible using *ICIM mapping* software (version 4.2.53) [118] in constructed populations, including RILs or DHLs. No direct software is available to detect such interactions in natural populations, including germplasm collections. Hence, in-house scripts were developed to look for loci interactions for various traits, as has been reported for yield and Karnal bunt resistance in wheat [165,166]. Hence, in the future, epistatic QTLs should be examined to capture hidden variance and define missing links in seed longevity research.

The discussion above indicates that the most important steps to discover the genetic determinants of seed longevity are population development, genotyping, intensive phenotyping, and computational analysis. All of these steps are expensive. Consequently, scientists, breeders, seed technologists, physiologists, molecular biologists, and genebank curators must exchange the germplasm developed by them in their respective projects to accelerate genetic research on seed longevity. Table 1 provides some examples of germplasm resources of different plant species that can be exploited to hasten research on seed longevity.

With the arrival of clustered regularly interspaced short palindromic repeats (CRISPR)/CRISPR-associated protein-9 (Cas9) and its increasing popularity in the plant research community [177], substantial improvements in our understanding of the genetic mechanisms of important biological functions in model and crop plants are imminent. Much detailed information is already available, as previously reviewed for horticultural plants [177]. However, there are no reports where seed longevity was targeted by this tool in any plant species. Thus, we also propose that the seed longevity genes identified and discussed in the preceding paragraphs in a range of plant species can be a useful target for CRISPR/Cas9 scientists for future research. Next-generation sequencing platforms provide new insights into the genome, transcriptome, and epigenome of plants, providing an advanced understanding of functional genes [178]. We propose that a time-course transcriptomic analysis can be used to identify transcriptional changes during aging treatments at key intervals in response to stress, as recently demonstrated. Reference [179] studied salt stress in wheat via Massive Analysis of cDNA 3′-ends (MACE). MACE sequencing can be used as a substitute for regular RNA sequencing, in which a single fragment represents one transcript [180]. Digital and strand-specific outputs of MACE with increased accuracy of expression data have provided high-resolution mapping to detect genes with moderate expression levels and short transcripts [180,181]. Similar to CRISPR/Cas9, MACE sequencing to detect the genetic control of seed longevity has yet to be undertaken.

In conclusion, genetic advances have been successful in establishing genetic determinants of seed longevity in wheat, barley, rice, maize, and other crop plants, in addition to horticultural plants, including tomato and lettuce. New techniques, including CRISPR/Cas9, RNA sequencing, and MACE sequencing, remain to be applied in the seed science research.

## Figures and Tables

**Table 1:** Germplasm collections with genotype data for future research on seed longevity of various crops.

Sr. No.	Plant Species	Number of Accessions	Genotyping Platform	References
1	Hexaploid wheat (*Triticum aestivum* L.)	>2500 accession in SeeDs of Discovery project	7180 genotyping by sequencing (GBS) SNPs	[167,168]
2	Durum wheat (*Triticum durum* Desf.)	(i) 6280 RILs (50 interconnected families constituting a nested association mapping population (NAM)) (ii) 1336 genotypes (from 25 families constituting a NAM)	(i) 13,000 SNPs from Infinium 15K Ultra HD chip (ii) 5398 SNPs from Illumina Infinium iSelect HD 9k chip	(i) [169] (ii) [170]
3	Sorghum (*Sorghum bicolor* (L.) Moench.)	971 world wide accessions	GBS SNPs	[171]
4	Rice (*Oryza sativa* L.)	1568 inbred varieties	700,000 high density rice array SNPs	[172]
5	Tomato (*Solanum lycopersicum* L.)	163 accesions, 291 accesions and 402 accesions	5995 SNPs, 9013 SNPs and 2014,488 SNPs, respectively	[173]
6	Pepper (*Capsicum* spp.)	10,038 genebank accession	GBS SNPs	[174]
7	Soybean (*Glycine max* L.)	(i) 421 accesions (ii) 305 accessions	(i) 1536 SNPs (ii) 37,573 SNPs	(i) [175] (ii) [176]

## Abbreviations

1-Cys Prx	1-cys peroxiredoxin and/or PER1
AA	artificial ageing
ABA	abscisic acid
AKR1	aldo-ketoreductases
Ale	Aleurain
AM	association mapping
AS	alternative splicing
ASPG1	ASPARTIC PROTEASE IN GUARD CELL 1
ATM	ATAXIA TELANGIECTASIA MUTATED (ATM)
ATR	RAD3-RELATED
BIL	backcross inbred lines
BR	brassinosteroid
BSA-seq	bulked segregant analysis through whole genome sequencing
Cas-9	CRISPR-associated protein-9
CDT	controlled deterioration test
CRISPR	clustered regularly interspaced short palindromic repeats
CSSL	chromosome segment substitution lines
DArT	diversity array technology
DEG	differentially expressed genes
DH	doubled haploid
DREB	dehydration responsive element binding protein
EIN2	ETHYLENE-INSENSITIVE 2-like
GA	gibberellins
ERE	ethylene-responsive element
GAAS	germination ability after storage
GBS	genotyping by sequencing (GBS
GO	gene ontology
GWAS	genome wide association study
HSP	heat shock proteins
IF_2_	immortalized F_2_
IAA	indole-3-acetic acid [IAA]
ITMI/MP	International Triticeae Mapping Initiative” mapping population
LACS2	long-chain acyl-CoA synthetase 2
LEA	late embryogenesis abundant
LG	linkage groups
LOX	lipoxygenase
MACE	massive analysis of cDNA 3′-ends
miRNA	microRNA
mQTL	meta-QTL
NADP-ME	NADP-dependent malic enzyme
NAM	nested association mapping
NIL	near isogenic line
NS	null segregant
OS	oligosaccharides
OsALDH7	rice aldehyde dehydrogenase
Per2	peroxidase
PIMT1	protein L-isoaspartyle methyltransferase
PPR	pentatricopeptide repeat
QTL	quantitative trait loci
RAB18	responsive to abscisic acid 18
RFO	raffinose family oligosaccharide
Rluc	Rennila LUCIFERASE reporter
RFO	raffinose series oligosaccharides
RIL	recombinant inbred population
RNA-seq	RNA sequence
SNP	single nucleotide polymorphism
SSP	seed storage proteins
SSR	simple sequence repeats
TIP	tonoplast intrinsic proteins
TPP	trehalose-6-phosphate phosphatase
Vps29	vacuolar protein sorting 29
Wip	wound-induced protein

## References

[1] Padulosi S. (1999). Final Report: Conservation and Use of Underutilized Mediterranean Species.

[2] Levin S.A. (2013). Encyclopedia of Biodiversity.

[3] Arif M.A.R., Börner A. (2019). Mapping of QTL associated with seed longevity in durum wheat (Triticum durum Desf.). J. Appl. Genet..

[4] Börner A., Khlestkina E.K., Chebotar S., Nagel M., Arif M.A.R., Neumann K., Kobiljski B., Lohwasser U., Röder M.S. (2012). Molecular markers in management of ex situ PGR–A case study. J. Biosci..

[5] Sano N., Rajjou L., North H.M., Debeaujon I., Marion-Poll A., Seo M. (2016). Staying alive: Molecular aspects of seed longevity. Plant Cell Physiol..

[6] Barton L.V. (1961). Seed Preservation and Longevity.

[7] Rehman Arif M.A., Börner A. (2020). An SNP based GWAS analysis of seed longevity in wheat. Cereal Res. Commun..

[8] Roberts E. (1961). Viability of cereal seed for brief and extended periods. Ann. Bot..

[9] McDonald M. (1999). Seed deterioration: Physiology, repair and assessment. Seed Sci. Technol..

[10] Coolbear P. (2020). Mechanisms of seed deterioration. Seed Quality.

[11] McGee D.C. (2000). Pathology of seed deterioration. Genet. Improv. Seed Qual..

[12] Clerkx E.J., El-Lithy M.E., Vierling E., Ruys G.J., Blankestijn-De Vries H., Groot S.P., Vreugdenhil D., Koornneef M. (2004). Analysis of natural allelic variation of Arabidopsis seed germination and seed longevity traits between the accessions Landsberg erecta and Shakdara, using a new recombinant inbred line population. Plant Physiol..

[13] Wettlaufer S.H., Leopold A.C. (1991). Relevance of Amadori and Maillard products to seed deterioration. Plant Physiol..

[14] Priestley D.A. (1986). Seed Aging: Implications for Seed Storage and Persistence in the Soil.

[15] Bartosz G. (1981). Non-specific reactions: Molecular basis for ageing. J. Theor. Biol..

[16] McDonald M. (1985). Physical Seed Quality of Soybean. Seed Sci. Technol..

[17] Harrington J.F. (1960). Germination of seeds from carrot, lettuce, and pepper plants grown under severe nutrient deficiencies. Hilgardia.

[18] Haferkamp M.E., Smith L., Nilan R. (1953). Studies on Aged Seeds I Relation of Age of Seed to Germination and Longevity 1. Agron. J..

[19] Justice O.L., Bass L.N. (1978). Principles and Practices of Seed Storage.

[20] Kranner I., Minibayeva F.V., Beckett R.P., Seal C.E. (2010). What is stress? Concepts, definitions and applications in seed science. New Phytol..

[21] Davies M.J. (2005). The oxidative environment and protein damage. Biochim. Et Biophys. Acta (BBA)-Proteins Proteom..

[22] Bailly C., El-Maarouf-Bouteau H., Corbineau F. (2008). From intracellular signaling networks to cell death: The dual role of reactive oxygen species in seed physiology. Comptes Rendus Biol..

[23] Rajjou L., Lovigny Y., Groot S.P., Belghazi M., Job C., Job D. (2008). Proteome-wide characterization of seed aging in Arabidopsis: A comparison between artificial and natural aging protocols. Plant Physiol..

[24] Almoguera C., Prieto-Dapena P., Díaz-Martín J., Espinosa J.M., Carranco R., Jordano J. (2009). The HaDREB2 transcription factor enhances basal thermotolerance and longevity of seeds through functional interaction with HaHSFA9. BMC Plant Biol..

[25] Kibinza S., Vinel D., Côme D., Bailly C., Corbineau F. (2006). Sunflower seed deterioration as related to moisture content during ageing, energy metabolism and active oxygen species scavenging. Physiol. Plant..

[26] Sivori E., Nakayama F., Cigliano E. (1968). Germination of Achira seed (*Canna* sp.) approximately 550 years old. Nature.

[27] Shen-Miller J., Mudgett M.B., Schopf J.W., Clarke S., Berger R. (1995). Exceptional seed longevity and robust growth: Ancient sacred lotus from China. Am. J. Bot..

[28] Harrington J.F., Kozlowski T. (1972). Seed storage and longevity. Seed Biol..

[29] Nagel M., Börner A. (2010). The longevity of crop seeds stored under ambient conditions. Seed Sci. Res..

[30] Copeland L.O., McDonald M.F. (2012). Principles of Seed Science and Technology.

[31] Roberts E., Ellis R. (1977). Prediction of seed longevity at sub-zero temperatures and genetic resources conservation. Nature.

[32] Clerkx E.J., Vries H.B.-D., Ruys G.J., Groot S.P., Koornneef M. (2003). Characterization of green seed, an enhancer of abi3-1 in Arabidopsis that affects seed longevity. Plant Physiol..

[33] Contreras S., Bennett M.A., Metzger J.D., Tay D., Nerson H. (2009). Red to far-red ratio during seed development affects lettuce seed germinability and longevity. HortScience.

[34] Contreras S., Bennett M.A., Metzger J.D., Tay D. (2008). Maternal light environment during seed development affects lettuce seed weight, germinability, and storability. HortScience.

[35] Bewley J.D., Black M. (2013). Seeds: Physiology of Development and Germination.

[36] Arif M.A.R. (2012). Seed Longevity and Dormancy in Wheat (*Triticum aestivum* L.)-Phenotypic Variation and Genetic Mapping. Ph.D. Thesis.

[37] Fan S. (1963). On “Fan Shengzhi Shu”: An Agriculturistic Book of China Written in the First Century BC.

[38] Weiss M.G., Wentz J.B. (1937). Effect of luteus genes on longevity of seed in maize. J. Am. Soc. Agron..

[39] Lindstrom E. (1943). Genetic relations of inbred lines of corn. Rep. Agrie. Res. Part.

[40] Lindstrom E. (1942). Inheritance of seed longevity in maize inbreds and hybrids. Genetics.

[41] RAO A.P., Fleming A. (1979). Cytoplasmic–Genotypic Influences on Seed Viability in a Maize Inbred. Can. J. Plant Sci..

[42] Haber E. (1950). Longevity of the seed of sweet corn inbreds and hybrids. Proc. Am. Soc. Hortic.Sci..

[43] Scott G.E. (1981). Improvement for Accelerated Aging Response of Seed in Maize Populations 1. Crop Sci..

[44] Van der Mey J., Kilpatrick R., Smit I. (1982). The germination of wheat and oat seed stored at Bethlehem, Republic of South Africa, 1956–1981. Cereal Res. Commun..

[45] Arif M.A.R., Nagel M., Lohwasser U., Börner A. (2017). Genetic architecture of seed longevity in bread wheat (*Triticum aestivum* L.). J. Biosci..

[46] Mackay D., Tonkin J. (1967). Investigations in crop seed longevity. I. An analysis of long-term experiments, with special reference to the influence of species, cultivar, provenance and season. J. Natl. Inst. Agric. Bot..

[47] Flood R. (1978). Contribution of impermeable seed to longevity in Trifolium subterraneum (subterranean clover). Seed Sci. Technol..

[48] Van der Maesen L. (1984). Seed storage, viability and rejuvenation. Genetic Resources and Their Exploitation—Chickpeas, Faba Beans and Lentils.

[49] Starzinger E., EK S., SH W. (1982). An observation on the relationship of soybean seed coat colour to viability maintenance. Seed Sci. Technol..

[50] Nakayama R., Saito K. (1980). Diallel analysis of the longevity of seeds in kidney beans [Phaseolus vulgaris]. Bull. Fac. Agric. Hirosaki Univ..

[51] Kueneman E. (1983). Genetic Control of Seed Longevity in Soybeans 1. Crop Sci..

[52] Nagel M., Vogel H., Landjeva S., Buck-Sorlin G., Lohwasser U., Scholz U., Börner A. (2009). Seed conservation in ex situ genebanks—Genetic studies on longevity in barley. Euphytica.

[53] Nagel M., Rosenhauer M., Willner E., Snowdon R.J., Friedt W., Börner A. (2011). Seed longevity in oilseed rape (*Brassica napus* L.)–genetic variation and QTL mapping. Plant Genet. Resour..

[54] Nagel M., Rehman-Arif M., Rosenhauer M., Börner A. (2010). Longevity of seeds-intraspecific differences in the Gatersleben genebank collections. Tagungsband.

[55] Bentsink L., Alonso-Blanco C., Vreugdenhil D., Tesnier K., Groot S.P., Koornneef M. (2000). Genetic analysis of seed-soluble oligosaccharides in relation to seed storability of Arabidopsis. Plant Physiol..

[56] de Souza Vidigal D., Willems L., van Arkel J., Dekkers B.J., Hilhorst H.W., Bentsink L. (2016). Galactinol as marker for seed longevity. Plant Sci..

[57] Clerkx E.J., Blankestijn-De Vries H., Ruys G.J., Groot S.P., Koornneef M. (2004). Genetic differences in seed longevity of various Arabidopsis mutants. Physiol. Plant..

[58] Sattler S.E., Gilliland L.U., Magallanes-Lundback M., Pollard M., DellaPenna D. (2004). Vitamin E is essential for seed longevity and for preventing lipid peroxidation during germination. Plant Cell.

[59] Gerna D., Arc E., Holzknecht M., Roach T., Jansen-Dürr P., Weiss A.K., Kranner I. (2021). AtFAHD1a: A New Player Influencing Seed Longevity and Dormancy in Arabidopsis?. Int. J. Mol. Sci..

[60] Ogé L., Bourdais G., Bove J., Collet B., Godin B., Granier F., Boutin J.-P., Job D., Jullien M., Grappin P. (2008). Protein repair L-isoaspartyl methyltransferase1 is involved in both seed longevity and germination vigor in Arabidopsis. Plant Cell.

[61] Hundertmark M., Buitink J., Leprince O., Hincha D.K. (2011). The reduction of seed-specific dehydrins reduces seed longevity in Arabidopsis thaliana. Seed Sci. Res..

[62] Nguyen T.-P., Keizer P., van Eeuwijk F., Smeekens S., Bentsink L. (2012). Natural variation for seed longevity and seed dormancy are negatively correlated in Arabidopsis. Plant Physiol..

[63] Bueso E., Ibañez C., Sayas E., Muñoz-Bertomeu J., Gonzalez-Guzmán M., Rodriguez P.L., Serrano R. (2014). A forward genetic approach in Arabidopsis thaliana identifies a RING-type ubiquitin ligase as a novel determinant of seed longevity. Plant Sci..

[64] Bueso E., Muñoz-Bertomeu J., Campos F., Brunaud V., Martínez L., Sayas E., Ballester P., Yenush L., Serrano R. (2014). ARABIDOPSIS THALIANA HOMEOBOX25 uncovers a role for Gibberellins in seed longevity. Plant Physiol..

[65] He H., de Souza Vidigal D., Snoek L.B., Schnabel S., Nijveen H., Hilhorst H., Bentsink L. (2014). Interaction between parental environment and genotype affects plant and seed performance in Arabidopsis. J. Exp. Bot..

[66] Mao Z., Sun W. (2015). Arabidopsis seed-specific vacuolar aquaporins are involved in maintaining seed longevity under the control of ABSCISIC ACID INSENSITIVE 3. J. Exp. Bot..

[67] Nguyen T.-P., Cueff G., Hegedus D.D., Rajjou L., Bentsink L. (2015). A role for seed storage proteins in Arabidopsis seed longevity. J. Exp. Bot..

[68] Waterworth W.M., Footitt S., Bray C.M., Finch-Savage W.E., West C.E. (2016). DNA damage checkpoint kinase ATM regulates germination and maintains genome stability in seeds. Proc. Natl. Acad. Sci. USA.

[69] Chen H.H., Chu P., Zhou Y.L., Ding Y., Li Y., Liu J., Jiang L.w., Huang S.Z. (2016). Ectopic expression of NnPER1, a Nelumbo nucifera 1-cysteine peroxiredoxin antioxidant, enhances seed longevity and stress tolerance in Arabidopsis. Plant J..

[70] Sano N., Kim J.-S., Onda Y., Nomura T., Mochida K., Okamoto M., Seo M. (2017). RNA-Seq using bulked recombinant inbred line populations uncovers the importance of brassinosteroid for seed longevity after priming treatments. Sci. Rep..

[71] Shen W., Yao X., Ye T., Ma S., Liu X., Yin X., Wu Y. (2018). Arabidopsis aspartic protease ASPG1 affects seed dormancy, seed longevity and seed germination. Plant Cell Physiol..

[72] Boussardon C., Martin-Magniette M.-L., Godin B., Benamar A., Vittrant B., Citerne S., Mary-Huard T., Macherel D., Rajjou L., Budar F. (2019). Novel cytonuclear combinations modify Arabidopsis thaliana seed physiology and vigor. Front. Plant Sci..

[73] Tsujimura M., Terachi T. (2018). Cytoplasmic Genome. The Allium Genomes.

[74] Yazdanpanah F., Maurino V.G., Mettler-Altmann T., Buijs G., Bailly M., Karimi Jashni M., Willems L., Sergeeva L.I., Rajjou L., Hilhorst H.W. (2019). NADP-MALIC ENZYME 1 affects germination after seed storage in Arabidopsis thaliana. Plant Cell Physiol..

[75] Durand T.C., Cueff G., Godin B., Valot B., Clément G., Gaude T., Rajjou L. (2019). Combined proteomic and metabolomic profiling of the Arabidopsis thaliana vps29 mutant reveals pleiotropic functions of the retromer in seed development. Int. J. Mol. Sci..

[76] Renard J., Niñoles R., Martínez-Almonacid I., Gayubas B., Mateos-Fernández R., Bissoli G., Bueso E., Serrano R., Gadea J. (2020). Identification of novel seed longevity genes related to oxidative stress and seed coat by genome-wide association studies and reverse genetics. Plant Cell Environ..

[77] Renard J., Martínez-Almonacid I., Sonntag A., Molina I., Moya-Cuevas J., Bissoli G., Muñoz-Bertomeu J., Faus I., Niñoles R., Shigeto J. (2020). PRX2 and PRX25, peroxidases regulated by COG1, are involved in seed longevity in Arabidopsis. Plant Cell Environ..

[78] Renard J., Martínez-Almonacid I., Queralta Castillo I., Sonntag A., Hashim A., Bissoli G., Campos L., Muñoz-Bertomeu J., Niñoles R., Roach T. (2021). Apoplastic lipid barriers regulated by conserved homeobox transcription factors extend seed longevity in multiple plant species. New Phytol..

[79] Miura K., Lin S., Yano M., Nagamine T. (2002). Mapping quantitative trait loci controlling seed longevity in rice (*Oryza sativa* L.). Theor. Appl. Genet..

[80] Sasaki K., Fukuta Y., Sato T. (2005). Mapping of quantitative trait loci controlling seed longevity of rice (*Oryza sativa* L.) after various periods of seed storage. Plant Breed..

[81] Zeng D., Guo L., Xu Y., Yasukumi K., Zhu L., Qian Q. (2006). QTL analysis of seed storability in rice. Plant Breed..

[82] Shigemune A., Miura K., Sasahara H., Goto A., Yoshida T. (2008). Role of maternal tissues in qLG-9 control of seed longevity in rice (*Oryza sativa* L.). Breed. Sci..

[83] Xue Y., Zhang S., Yao Q., Peng R., Xiong A., Li X., Zhu W., Zhu Y., Zha D. (2008). Identification of quantitative trait loci for seed storability in rice (*Oryza sativa* L.). Euphytica.

[84] Sasaki K., Takeuchi Y., Miura K., Yamaguchi T., Ando T., Ebitani T., Higashitani A., Yamaya T., Yano M., Sato T. (2015). Fine mapping of a major quantitative trait locus, qLG-9, that controls seed longevity in rice (*Oryza sativa* L.). Theor. Appl. Genet..

[85] Shin J.-H., Kim S.-R., An G. (2008). Rice Aldehyde Dehydrogenase7 Is Needed for Seed Maturation and Viability. Plant Physiol..

[86] Jiang W., Lee J., Jin Y.-M., Qiao Y., Piao R., Jang S.M., Woo M.-O., Kwon S.-W., Liu X., Pan H.-Y. (2011). Identification of QTLs for seed germination capability after various storage periods using two RIL populations in rice. Mol. Cells.

[87] Li L., Lin Q., Liu S., Liu X., Wang W., Hang N.T., Liu F., Zhao Z., Jiang L., Wan J. (2012). Identification of quantitative trait loci for seed storability in rice (*Oryza sativa* L.). Plant Breed..

[88] Long Q., Zhang W., Wang P., Shen W., Zhou T., Liu N., Wang R., Jiang L., Huang J., Wang Y. (2013). Molecular genetic characterization of rice seed lipoxygenase 3 and assessment of its effects on seed longevity. J. Plant Biol..

[89] Xu H., Wei Y., Zhu Y., Lian L., Xie H., Cai Q., Chen Q., Lin Z., Wang Z., Xie H. (2015). Antisense suppression of LOX3 gene expression in rice endosperm enhances seed longevity. Plant Biotechnol. J..

[90] Huang J., Cai M., Long Q., Liu L., Lin Q., Jiang L., Chen S., Wan J. (2014). OsLOX2, a rice type I lipoxygenase, confers opposite effects on seed germination and longevity. Transgenic Res..

[91] Hang N.T., Lin Q., Liu L., Liu X., Liu S., Wang W., Li L., He N., Liu Z., Jiang L. (2015). Mapping QTLs related to rice seed storability under natural and artificial aging storage conditions. Euphytica.

[92] Wei Y., Xu H., Diao L., Zhu Y., Xie H., Cai Q., Wu F., Wang Z., Zhang J., Xie H. (2015). Protein repair L-isoaspartyl methyltransferase 1 (PIMT1) in rice improves seed longevity by preserving embryo vigor and viability. Plant Mol. Biol..

[93] Li G., Na Y.-W., Kwon S.-W., Park Y.-J. (2014). Association analysis of seed longevity in rice under conventional and high-temperature germination conditions. Plant Syst. Evol..

[94] Lee J.-S., Kwak J., Yoon M.-R., Lee J.-S., Hay F.R. (2017). Contrasting tocol ratios associated with seed longevity in rice variety groups. Seed Sci. Res..

[95] Lee J.-S., Kwak J., Cho J.-H., Chebotarov D., Yoon M.-R., Lee J.-S., Hamilton N.R.S., Hay F.R. (2019). A high proportion of beta-tocopherol in vitamin E is associated with poor seed longevity in rice produced under temperate conditions. Plant Genet. Resour..

[96] Nisarga K.N., Vemanna R.S., Chandrashekar B.K., Rao H., Vennapusa A.R., Narasimaha A., Makarla U., Basavaiah M.R. (2017). Aldo-ketoreductase 1 (AKR1) improves seed longevity in tobacco and rice by detoxifying reactive cytotoxic compounds generated during ageing. Rice.

[97] Zhou Y., Zhou S., Wang L., Wu D., Cheng H., Du X., Mao D., Zhang C., Jiang X. (2020). miR164c and miR168a regulate seed vigor in rice. J. Integr. Plant Biol..

[98] Liu W., Pan X., Li Y., Duan Y., Min J., Liu S., Sheng X., Li X. (2018). Detection and validation of QTL s associated with seed longevity in rice (*Oryza sativa* L.). Plant Breed..

[99] Yan S., Huang W., Gao J., Fu H., Liu J. (2018). Comparative metabolomic analysis of seed metabolites associated with seed storability in rice (*Oryza sativa* L.) during natural aging. Plant Physiol. Biochem..

[100] Lee J.-S., Velasco-Punzalan M., Pacleb M., Valdez R., Kretzschmar T., McNally K.L., Ismail A.M., Cruz P.C.S., Sackville Hamilton N.R., Hay F.R. (2019). Variation in seed longevity among diverse Indica rice varieties. Ann. Bot..

[101] Raquid R., Kohli A., Reinke R., Dionisio-Sese M., Kwak J., Chebotarov D., Mo Y., Lee J.-S. (2021). Genetic factors enhancing seed longevity in tropical japonica rice. Curr. Plant Biol..

[102] Wu F., Luo X., Wang L., Wei Y., Li J., Xie H., Zhang J., Xie G. (2021). Genome-Wide Association Study Reveals the QTLs for Seed Storability in World Rice Core Collections. Plants.

[103] Zhao M., Hu B., Fan Y., Ding G., Yang W., Chen Y., Chen Y., Xie J., Zhang F. (2021). Identification, Analysis, and Confirmation of Seed Storability-Related Loci in Dongxiang Wild Rice (Oryza rufipogon Griff.). Genes.

[104] Lundqvist U. (1997). New and revised descriptions of barley genes. Barley Genet. Newslett..

[105] Sun S., Yu J.-P., Chen F., Zhao T.-J., Fang X.-H., Li Y.-Q., Sui S.-F. (2008). TINY, a dehydration-responsive element (DRE)-binding protein-like transcription factor connecting the DRE-and ethylene-responsive element-mediated signaling pathways in Arabidopsis. J. Biol. Chem..

[106] Zareie R., Melanson D.L., Murphy P.J. (2002). Isolation of fungal cell wall degrading proteins from barley (*Hordeum vulgare* L.) leaves infected with Rhynchosporium secalis. Mol. Plant-Microbe Interact..

[107] Nagel M., Kranner I., Neumann K., Rolletschek H., Seal C.E., Colville L., Fernández-Marín B., Börner A. (2015). Genome-wide association mapping and biochemical markers reveal that seed ageing and longevity are intricately affected by genetic background and developmental and environmental conditions in barley. Plant Cell Environ..

[108] Wozny D., Kramer K., Finkemeier I., Acosta I.F., Koornneef M. (2018). Genes for seed longevity in barley identified by genomic analysis on near isogenic lines. Plant Cell Environ..

[109] Puchta M., Groszyk J., Małecka M., Koter M.D., Niedzielski M., Rakoczy-Trojanowska M., Boczkowska M. (2021). Barley Seeds miRNome Stability during Long-Term Storage and Aging. Int. J. Mol. Sci..

[110] Landjeva S., Lohwasser U., Börner A. (2010). Genetic mapping within the wheat D genome reveals QTL for germination, seed vigour and longevity, and early seedling growth. Euphytica.

[111] Arif M.R., Nagel M., Neumann K., Kobiljski B., Lohwasser U., Börner A. (2012). Genetic studies of seed longevity in hexaploid wheat using segregation and association mapping approaches. Euphytica.

[112] Li W., Faris J., Chittoor J., Leach J., Hulbert S., Liu D., Chen P., Gill B. (1999). Genomic mapping of defense response genes in wheat. Theor. Appl. Genet..

[113] Quarrie S., Steed A., Calestani C., Semikhodskii A., Lebreton C., Chinoy C., Steele N., Pljevljakusić D., Waterman E., Weyen J. (2005). A high-density genetic map of hexaploid wheat (*Triticum aestivum* L.) from the cross Chinese Spring× SQ1 and its use to compare QTLs for grain yield across a range of environments. Theor. Appl. Genet..

[114] Sourdille P., Tixier M., Charmet G., Gay G., Cadalen T., Bernard S., Bernard M. (2000). Location of genes involved in ear compactness in wheat (Triticum aestivum) by means of molecular markers. Mol. Breed..

[115] Agacka-Mołdoch M., Arif M.A.R., Lohwasser U., Doroszewska T., Qualset C.O., Börner A. (2016). The inheritance of wheat grain longevity: A comparison between induced and natural ageing. J. Appl. Genet..

[116] Arif M.A.R., Shokat S., Plieske J., Ganal M., Lohwasser U., Chesnokov Y.V., Kocherina N.V., Kulwal P., Kumar N., McGuire P.E. (2021). A SNP-based genetic dissection of versatile traits in bread wheat (*Triticum aestivum* L.). Plant J..

[117] Arif M.A.R., Agacka-Mołdoch M., Qualset C.O., Börner A. (2022). Mapping of additive and epistatic QTLs linked to seed longevity in bread wheat (*Triticum aestivum* L.). Cereal Res. Commun.

[118] Meng L., Li H., Zhang L., Wang J. (2015). QTL IciMapping: Integrated software for genetic linkage map construction and quantitative trait locus mapping in biparental populations. Crop J..

[119] Kolodziej M.C., Singla J., Sánchez-Martín J., Zbinden H., Šimková H., Karafiátová M., Doležel J., Gronnier J., Poretti M., Glauser G. (2021). A membrane-bound ankyrin repeat protein confers race-specific leaf rust disease resistance in wheat. Nat. Commun..

[120] Yan J., Yao Y., Hong S., Yang Y., Shen C., Zhang Q., Zhang D., Zou T., Yin P. (2019). Delineation of pentatricopeptide repeat codes for target RNA prediction. Nucleic Acids Res..

[121] Figueiredo J., Sousa Silva M., Figueiredo A. (2018). Subtilisin-like proteases in plant defence: The past, the present and beyond. Mol. Plant Pathol..

[122] Reinbothe C., Springer A., Samol I., Reinbothe S. (2009). Plant oxylipins: Role of jasmonic acid during programmed cell death, defence and leaf senescence. FEBS J..

[123] Ohmiya A. (2009). Carotenoid cleavage dioxygenases and their apocarotenoid products in plants. Plant Biotechnol..

[124] Saibi W., Zouari N., Masmoudi K., Brini F. (2016). Role of the durum wheat dehydrin in the function of proteases conferring salinity tolerance in Arabidopsis thaliana transgenic lines. Int. J. Biol. Macromol..

[125] Sgarbi C., Malbrán I., Saldúa L., Lori G.A., Lohwasser U., Arif M.A.R., Börner A., Yanniccari M., Castro A.M. (2021). Mapping Resistance to Argentinean Fusarium (Graminearum) Head Blight Isolates in Wheat. Int. J. Mol. Sci..

[126] Zuo J., Liu J., Gao F., Yin G., Wang Z., Chen F., Li X., Xu J., Chen T., Li L. (2018). Genome-wide linkage mapping reveals qtls for seed vigor-related traits under artificial aging in common wheat (*Triticum aestivum* ). Front. Plant Sci..

[127] Zuo J.H., Chen F.Y., Li X.Y., Xia X.C., Cao H., Liu J.D., Liu Y.X. (2020). Genome-wide association study reveals loci associated with seed longevity in common wheat (*Triticum aestivum* L.). Plant Breed..

[128] Wang S., Wong D., Forrest K., Allen A., Chao S., Huang B.E., Maccaferri M., Salvi S., Milner S.G., Cattivelli L. (2014). Characterization of polyploid wheat genomic diversity using a high-density 90 000 single nucleotide polymorphism array. Plant Biotechnol. J..

[129] Wu X., Ning F., Hu X., Wang W. (2017). Genetic modification for improving seed vigor is transitioning from model plants to crop plants. Front. Plant Sci..

[130] Shi H., Guan W., Shi Y., Wang S., Fan H., Yang J., Chen W., Zhang W., Sun D., Jing R. (2020). QTL mapping and candidate gene analysis of seed vigor-related traits during artificial aging in wheat (*Triticum aestivum*). Sci. Rep..

[131] Revilla P., Velasco P., Malvar R.A., Cartea M.E., Ordás A. (2006). Variability among maize (*Zea mays* L.) inbred lines for seed longevity. Genet. Resour. Crop Evol..

[132] Revilla P., Butrón A., Rodríguez V., Malvar R., Ordás A. (2009). Identification of genes related to germination in aged maize seed by screening natural variability. J. Exp. Bot..

[133] Liu J.-B., Fu Z.-Y., Xie H.-L., Hu Y.-M., Liu Z.-H., Duan L.-J., Xu S.-Z., Tang J.-H. (2011). Identification of QTLs for maize seed vigor at three stages of seed maturity using a RIL population. Euphytica.

[134] Han Z., Ku L., Zhang Z., Zhang J., Guo S., Liu H., Zhao R., Ren Z., Zhang L., Su H. (2014). QTLs for seed vigor-related traits identified in maize seeds germinated under artificial aging conditions. PLoS ONE.

[135] Wang B., Zhang Z., Fu Z., Liu Z., Hu Y., Tang J. (2016). Comparative QTL analysis of maize seed artificial aging between an immortalized F2 population and its corresponding RILs. Crop J..

[136] Liu Y., Zhang H., Li X., Wang F., Lyle D., Sun L., Wang G., Wang J., Li L., Gu R. (2019). Quantitative trait locus mapping for seed artificial aging traits using an F2: 3 population and a recombinant inbred line population crossed from two highly related maize inbreds. Plant Breed..

[137] Li L., Wang F., Li X., Peng Y., Zhang H., Hey S., Wang G., Wang J., Gu R. (2019). Comparative analysis of the accelerated aged seed transcriptome profiles of two maize chromosome segment substitution lines. PLoS ONE.

[138] Han Q., Chen K., Yan D., Hao G., Qi J., Wang C., Dirk L.M., Bruce Downie A., Gong J., Wang J. (2020). ZmDREB2A regulates ZmGH3. 2 and ZmRAFS, shifting metabolism towards seed aging tolerance over seedling growth. Plant J..

[139] Guo X., Sun X., Liu S., Gong C., Feng C., Han X., Lv T., Zhou Y., Wang Z., Di H. (2021). Screening and application of SSR markers related to seed storability traits in maize (*Zea mays* L.). Genet. Resour. Crop Evol..

[140] Singh R., Raipuria R., Bhatia V., Rani A., Husain S., Chauhan D., Chauhan G., Mohapatra T. (2008). SSR markers associated with seed longevity in soybean. Seed Sci. Technol..

[141] Cregan P., Jarvik T., Bush A., Shoemaker R., Lark K., Kahler A., Kaya N., VanToai T., Lohnes D., Chung J. (1999). An integrated genetic linkage map of the soybean genome. Crop Sci..

[142] Singh R., Raipuria R., Bhatia V., Rani A., Husain S., Satyavathi C.T., Chauhan G., Mohapatra T. (2008). Identification of SSR markers associated with seed coat permeability and electrolyte leaching in soybean. Physiol. Mol. Biol. Plants.

[143] Naik S., Madhusudan K., Motagi B., Mugali S., Nadaf H. (2019). Molecular characterization of seed longevity and associated characters using SSR markers in soybean [*Glycine max* (L.) Merrill]. J. Pharmacogn. Phytochem..

[144] Hosamani J., Kumar M.A., Talukdar A., Lal S., Dadlani M. (2013). Molecular characterization and identification of candidate markers for seed longevity in soybean [*Glycine max* (L.) Merill]. Indian J. Genet..

[145] Dargahi H., Tanya P., Srinives P. (2014). Mapping of the genomic regions controlling seed storability in soybean (*Glycine max* L.). J. Genet..

[146] Pereira Lima J.J., Buitink J., Lalanne D., Rossi R.F., Pelletier S., Da Silva E.A.A., Leprince O. (2017). Molecular characterization of the acquisition of longevity during seed maturation in soybean. PLoS ONE.

[147] Zhang X., Hina A., Song S., Kong J., Bhat J.A., Zhao T. (2019). Whole-genome mapping identified novel “QTL hotspots regions” for seed storability in soybean (*Glycine max* L.). BMC Genom..

[148] Pawar P., Naik R., Deshmukh M., Satbhai R., Mohite S. (2019). Biochemical and molecuar marker based screening of seed longevity in soybean [*Glycine max* (L.) Merill]. Legume Res. Int. J..

[149] Wang R., Wu F., Xie X., Yang C. (2021). Quantitative Trait Locus Mapping of Seed Vigor in Soybean under −20 °C Storage and Accelerated Aging Conditions via RAD Sequencing. Curr. Issues Mol. Biol..

[150] Schwember A.R., Bradford K.J. (2010). Quantitative trait loci associated with longevity of lettuce seeds under conventional and controlled deterioration storage conditions. J. Exp. Bot..

[151] Agacka-Mołdoch M., Nagel M., Doroszewska T., Lewis R., Börner A. (2015). Mapping quantitative trait loci determining seed longevity in tobacco (*Nicotiana tabacum* L.). Euphytica.

[152] Xiao B., Drake K., Vontimitta V., Tong Z., Zhang X., Li M.Y., Leng X.D., Li Y., Lewis R.S. (2013). Location of genomic regions contributing to Phytophthora nicotianae resistance in tobacco cultivar Florida 301. Crop Sci..

[153] Agacka-Mołdoch M., Arif M.A.R., Lohwasser U., Doroszewska T., Lewis R.S., Börner A. (2021). QTL analysis of seed germination traits in tobacco (*Nicotiana tabacum* L.). J. Appl. Genet..

[154] Barbosa Batista T., Javier Fernandez G., Alexandre da Silva T., Maia J., Amaral da Silva E.A. (2020). Transcriptome analysis in osmo-primed tomato seeds with enhanced longevity by heat shock treatment. AoB Plants.

[155] Bizouerne E., Buitink J., Vu B.L., Vu J.L., Esteban E., Pasha A., Provart N., Verdier J., Leprince O. (2021). Gene co-expression analysis of tomato seed maturation reveals tissue-specific regulatory networks and hubs associated with the acquisition of desiccation tolerance and seed vigour. BMC Plant Biol..

[156] Graner A., Börner A. (2006). Quest for seed immortality is mission impossible. Nature.

[157] Zhu C., Gore M., Buckler E.S., Yu J. (2008). Status and prospects of association mapping in plants. Plant Genome.

[158] Jannink J.-L., Bink M.C., Jansen R.C. (2001). Using complex plant pedigrees to map valuable genes. Trends Plant Sci..

[159] Röder M.S., Korzun V., Wendehake K., Plaschke J., Tixier M.-H., Leroy P., Ganal M.W. (1998). A microsatellite map of wheat. Genetics.

[160] Arif M.R., Neumann K., Nagel M., Kobiljski B., Lohwasser U., Börner A. (2012). An association mapping analysis of dormancy and pre-harvest sprouting in wheat. Euphytica.

[161] Joosen R.V., Kodde J., Willems L.A., Ligterink W., van der Plas L.H., Hilhorst H.W. (2010). GERMINATOR: A software package for high-throughput scoring and curve fitting of Arabidopsis seed germination. Plant J..

[162] Nelson J.C. (1997). QGENE: Software for marker-based genomic analysis and breeding. Mol. Breed..

[163] Wang S., Basten C., Zeng Z., Raleigh N.C. (2012). Windows QTL Cartographer 2.5.

[164] Zhang Y.-W., Wen Y.-J., Dunwell J.M., Zhang Y.-M. (2020). QTL. gCIMapping. GUI v2. 0: An R software for detecting small-effect and linked QTLs for quantitative traits in bi-parental segregation populations. Comput. Struct. Biotechnol. J..

[165] Singh S., Sehgal D., Kumar S., Arif M., Vikram P., Sansaloni C., Fuentes-Dávila G., Ortiz C. (2020). GWAS revealed a novel resistance locus on chromosome 4D for the quarantine disease Karnal bunt in diverse wheat pre-breeding germplasm. Sci. Rep..

[166] Sehgal D., Autrique E., Singh R., Ellis M., Singh S., Dreisigacker S. (2017). Identification of genomic regions for grain yield and yield stability and their epistatic interactions. Sci. Rep..

[167] Singh S., Jighly A., Sehgal D., Burgueño J., Joukhadar R., Singh S., Sharma A., Vikram P., Sansaloni C., Govindan V. (2021). Direct introgression of untapped diversity into elite wheat lines. Nat. Food.

[168] Singh S., Vikram P., Sehgal D., Burgueño J., Sharma A., Singh S.K., Sansaloni C.P., Joynson R., Brabbs T., Ortiz C. (2018). Harnessing genetic potential of wheat germplasm banks through impact-oriented-prebreeding for future food and nutritional security. Sci. Rep..

[169] Kidane Y.G., Gesesse C.A., Hailemariam B.N., Desta E.A., Mengistu D.K., Fadda C., Pè M.E., Dell’Acqua M. (2019). A large nested association mapping population for breeding and quantitative trait locus mapping in Ethiopian durum wheat. Plant Biotechnol. J..

[170] Saade S., Maurer A., Shahid M., Oakey H., Schmöckel S.M., Negrão S., Pillen K., Tester M. (2016). Yield-related salinity tolerance traits identified in a nested association mapping (NAM) population of wild barley. Sci. Rep..

[171] Morris G.P., Ramu P., Deshpande S.P., Hash C.T., Shah T., Upadhyaya H.D., Riera-Lizarazu O., Brown P.J., Acharya C.B., Mitchell S.E. (2013). Population genomic and genome-wide association studies of agroclimatic traits in sorghum. Proc. Natl. Acad. Sci. USA.

[172] McCouch S.R., Wright M.H., Tung C.-W., Maron L.G., McNally K.L., Fitzgerald M., Singh N., DeClerck G., Agosto-Perez F., Korniliev P. (2016). Open access resources for genome-wide association mapping in rice. Nat. Commun..

[173] Zhao J., Sauvage C., Zhao J., Bitton F., Bauchet G., Liu D., Huang S., Tieman D.M., Klee H.J., Causse M. (2019). Meta-analysis of genome-wide association studies provides insights into genetic control of tomato flavor. Nat. Commun..

[174] Tripodi P., Rabanus-Wallace M.T., Barchi L., Kale S., Esposito S., Acquadro A., Schafleitner R., van Zonneveld M., Prohens J., Diez M.J. (2021). Global range expansion history of pepper (Capsicum spp.) revealed by over 10,000 genebank accessions. Proc. Natl. Acad. Sci. USA.

[175] Li Y.-H., Reif J.C., Hong H.-L., Li H.-H., Liu Z.-X., Ma Y.-S., Li J., Tian Y., Li Y.-F., Li W.-B. (2018). Genome-wide association mapping of QTL underlying seed oil and protein contents of a diverse panel of soybean accessions. Plant Sci..

[176] Do T.D., Vuong T.D., Dunn D., Clubb M., Valliyodan B., Patil G., Chen P., Xu D., Nguyen H.T., Shannon J.G. (2019). Identification of new loci for salt tolerance in soybean by high-resolution genome-wide association mapping. BMC Genom..

[177] Bhatta B.P., Malla S. (2020). Improving horticultural crops via CRISPR/Cas9: Current successes and prospects. Plants.

[178] Yadav P., Vaidya E., Rani R., Yadav N.K., Singh B., Rai P., Singh D. (2018). Recent perspective of next generation sequencing: Applications in molecular plant biology and crop improvement. Proc. Natl. Acad.Sci. India Sect. B Biol. Sci..

[179] Duarte-Delgado D., Dadshani S., Schoof H., Oyiga B.C., Schneider M., Mathew B., Léon J., Ballvora A. (2020). Transcriptome profiling at osmotic and ionic phases of salt stress response in bread wheat uncovers trait-specific candidate genes. BMC Plant Biol..

[180] Hrdlickova R., Toloue M., Tian B. (2017). RNA-Seq methods for transcriptome analysis. Wiley Interdiscip.Rev. RNA.

[181] Asmann Y.W., Klee E.W., Thompson E.A., Perez E.A., Middha S., Oberg A.L., Therneau T.M., Smith D.I., Poland G.A., Wieben E.D. (2009). 3’tag digital gene expression profiling of human brain and universal reference RNA using Illumina Genome Analyzer. BMC Genom..

